# Heterosubtypic Protections against Human-Infecting Avian Influenza Viruses Correlate to Biased Cross-T-Cell Responses

**DOI:** 10.1128/mBio.01408-18

**Published:** 2018-08-07

**Authors:** Min Zhao, Kefang Liu, Jiejian Luo, Shuguang Tan, Chuansong Quan, Shuijun Zhang, Yan Chai, Jianxun Qi, Yan Li, Yuhai Bi, Haixia Xiao, Gary Wong, Jianfang Zhou, Taijiao Jiang, Wenjun Liu, Hongjie Yu, Jinghua Yan, Yingxia Liu, Yuelong Shu, Guizhen Wu, Aiping Wu, George F. Gao, William J. Liu

**Affiliations:** a CAS Key Laboratory of Pathogenic Microbiology and Immunology, Institute of Microbiology, Chinese Academy of Sciences (CAS), Beijing, China; b Research Network of Immunity and Health (RNIH), Beijing Institutes of Life Science, University of Chinese Academy of Sciences, Beijing, China; c College of Laboratory Medicine and Life Sciences, Wenzhou Medical University, Wenzhou, China; d Key Laboratory of Medical Virology, Ministry of Health, National Institute for Viral Disease Control and Prevention, Chinese Center for Disease Control and Prevention (China CDC), Beijing, China; e Center for Systems Medicine, Institute of Basic Medical Sciences, Chinese Academy of Medical Sciences and Peking Union Medical College, Beijing, China; f Suzhou Institute of Systems Medicine, Suzhou, China; g CAS Key Laboratory of Protein and Peptide Pharmaceuticals, Institute of Biophysics, Chinese Academy of Sciences (CAS), Beijing, China; h Center for Influenza Research and Early-Warning (CASCIRE), Chinese Academy of Sciences, Beijing, China; i Shenzhen Key Laboratory of Pathogen and Immunity, State Key Discipline of Infectious Disease, Shenzhen Third People’s Hospital, Shenzhen, China; j Laboratory of Protein Engineering and Vaccine, Tianjin Institute of Industrial Biotechnology, Chinese Academy of Sciences, Tianjin, China; k Key Laboratory of Surveillance and Early Warning on Infectious Disease, Division of Infectious Disease, Chinese Center for Disease Control and Prevention, Beijing, China; Max Planck Institute for Infection Biology

**Keywords:** T-cell responses, avian influenza viruses, cross-reactivity, heterosubtypic protection

## Abstract

Against a backdrop of seasonal influenza virus epidemics, emerging avian influenza viruses (AIVs) occasionally jump from birds to humans, posing a public health risk, especially with the recent sharp increase in H7N9 infections. Evaluations of cross-reactive T-cell immunity to seasonal influenza viruses and human-infecting AIVs have been reported previously. However, the roles of influenza A virus-derived epitopes in the cross-reactive T-cell responses and heterosubtypic protections are not well understood; understanding those roles is important for preventing and controlling new emerging AIVs. Here, among the members of a healthy population presumed to have previously been infected by pandemic H1N1 (pH1N1), we found that pH1N1-specific T cells showed cross- but biased reactivity to human-infecting AIVs, i.e., H5N1, H6N1, H7N9, and H9N2, which correlates with distinct protections. Through a T-cell epitope-based phylogenetic analysis, the cellular immunogenic clustering expanded the relevant conclusions to a broader range of virus strains. We defined the potential key conserved epitopes required for cross-protection and revealed the molecular basis for the immunogenic variations. Our study elucidated an overall profile of cross-reactivity to AIVs and provided useful recommendations for broad-spectrum vaccine development.

## IMPORTANCE

We revealed preexisting but biased T-cell reactivity of pH1N1 influenza virus to human-infecting AIVs, which provided distinct protections. The cross-reactive T-cell recognition had a regular pattern that depended on the T-cell epitope matrix revealed via bioinformatics analysis. Our study elucidated an overall profile of cross-reactivity to AIVs and provided useful recommendations for broad-spectrum vaccine development.

## INTRODUCTION

Influenza viruses (IVs) continuously pose a threat to public health due to point mutations (antigenic drift) and reassortment events (antigenic shift), especially in the viral surface proteins hemagglutinin (HA) and neuraminidase (NA). In recent years, new influenza viruses have emerged, leading to occasional epidemics or even pandemics. In 2009, a novel H1N1 strain, 2009 pandemic influenza A (H1N1) virus (pH1N1), was first reported in La Gloria, Veracruz, Mexico (1). It quickly spread all over the world and is still circulating as one of the dominant seasonal influenza viruses in various countries (2). After several seasons of circulation, an overall high (up to 24%) incidence of pH1N1 exists among the populations of those countries (3). Meanwhile, different avian influenza viruses (AIVs) also emerged or reemerged, posing continuously threats to global health. From 2003 to 2015, 844 laboratory-confirmed human cases (with 449 deaths) of avian influenza A (H5N1) virus infection were officially reported to the World Health Organization (WHO) from 16 countries. Hundreds of human infection cases (with a mortality rate of 40%) were reported for avian influenza A (H7N9) virus since March 2013 (http://www.who.int/influenza). Additionally, H9N2, H6N1, and H5N6 have emerged, all of which are connected with poultry exposure (4–6). These AIVs pose a threat to human health, considering the circulation of the viruses in the live-poultry market (LPM) and among wild birds (7, 8).

Cellular immunity plays a key role in the control of influenza virus infection (9–13). CD4^+^ T cells can promote effective immunity by providing secondary signals for antibody (Ab) responses and produce cytokines upon infection (14), while cytotoxic CD8^+^ T cells can provide partial protection and reduce symptoms by promoting viral clearance (15). Infection with influenza A virus often provides heterosubtypic immunity against other subtypes (16). Although cross-subtype monoclonal antibodies (MAbs) against influenza viruses have been reported (17), heterosubtypic neutralizing antibody responses among the populations are rare (18). Influenza A viruses mainly express 14 proteins which are encoded by eight RNA segments (19). Cytotoxic T lymphocytes (CTLs) specific for influenza A viruses mostly target internal, nonglycosylated proteins, such as NP, M1, and PB1 (20), which are enriched with immunodominant CTL epitopes and markedly conserved among different strains compared to HA and NA. This implies an important role for virus-specific T cells in heterosubtypic immune responses. Influenza virus-specific cross-T-cell reactivities between seasonal influenza virus and a particular AIV, such as H5N1 or H7N9, have previously been investigated (10, 20–22). However, a systematic evaluation of cross-subtypic T-cell immunity profiles with respect to different human-infecting AIVs has not yet been performed, and the roles of influenza A virus-derived epitopes in the cross-reactive T-cell responses and heterosubtypic protection are not well understood.

Recent vaccine development efforts have emphasized universal protection against different influenza subtypes, which mainly depends on cross-T-cell reactivity in addition to heterologous antibodies (23). Increasing the magnitude of memory CD8^+^ T cells could provide better protection against heterosubtypic infections (24). It was reported that an effective vaccine from modified vaccinia virus Ankara (MVA) encoding the A/Panama/2007/2099 NP and M1 proteins drastically boosts CTL responses in phase 1 and phase 2a clinical trials in healthy adults. The protective efficacy of the elicited CTLs by the T-cell-based influenza vaccine can be confirmed by influenza challenge (25). Nevertheless, combined structural and functional studies based on influenza virus-derived T-cell epitopes demonstrated that minor mutation of an epitope can lead to a profound effect on the antigenicity of the peptide (26–28). Although the internal proteins targeted by these potential universal vaccines are quite conserved between different influenza virus subtypes, it is largely unknown whether minor variations in these T-cell epitopes would have an influence on protection.

Here, we evaluated preexisting T-cell responses to different human-infecting AIVs in a healthy cohort. The contribution of the cross-reactive T-cell responses to protection against AIVs was also determined in a mouse model. The underlying molecular basis was investigated using epitope-based phylogenetic trees and crystal structure determinations. The data revealed the cross-immune profile in major human-infecting subtypes of AIVs and pH1N1, providing a guide for the development of universal influenza vaccines.

## RESULTS

### Undetectable cross-antibody responses to AIVs.

**FIG 1:**
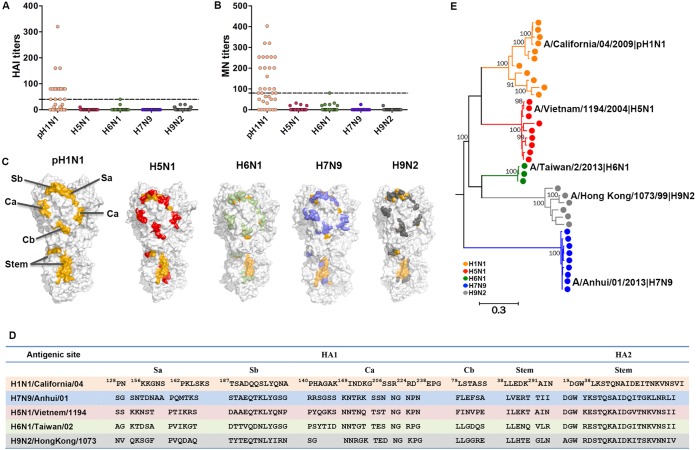
Humoral immune responses to pH1N1 and AIVs. (A and B) Humoral responses of healthy donors (*n* = 35) were detected by HAI assays (A) and MN assays (B) with A(H1N1)/California/4/2009, A(H5N1)/Vietnam/1194/2004, A(H6N1)/Taiwan/2/2013, A(H7N9)/Anhui/1/2013, or A(H9N2)/Hong Kong/1073/99. Dashed lines indicate the following cutoff values: 40 for the HAI assays and 80 for the MN assays. (C) Locations of the major antigenic sites for antibody binding of the HA protein are marked based on the structures of the pH1N1 HA protein (PDB code 4JTV), H5N1 HA (PDB code 3ZNK), H6N1 HA (PDB code 4YY9), H7N9 HA (PDB code 4KO1), and H9N2 HA (PDB code 1JSD). The mutant sites in the HA protein of each subtype in B-cell epitopes compared to pH1N1 HA protein are marked with different colors (H5N1 as red, H6N1 as green, H7N9 as blue, and H9N2 as dark gray). (D) Sequences of B cell epitopes are shown in the table. (E) The phylogenetic relationship of the representative human-infecting AIV strains and pH1N1 were analyzed by the maximum-likelihood method with MEGA 6. The names of the viruses we used in HAI and MN assays are denoted with black characters. HAI, HA inhibition; MN, microneutralization.

A population of healthy volunteers (*n* = 35) residing in Beijing, China, was recruited in 2014, and peripheral blood mononuclear cells (PBMCs) were collected from 30 of these volunteers (see Table S1 in the supplemental material). Their humoral responses against A(H1N1)/California/04/2009 and different human-infecting AIVs were tested through both HA inhibition (HAI) and microneutralization (MN) (29) assays. The results showed pH1N1-specific HAI titers (≥40) and MN titers (≥80) among 49% (17/35) and 60% (21/35) of the subjects, respectively (Fig. 1A and B). Among the 35 subjects, 23 were A(H1N1)/California/04 antibody positive (pH1N1 Ab^+^) by HAI or MN assay. This indicated a high ratio of previous pH1N1 exposure in the population. In contrast, no specific antibodies against A(H5N1)/Vietnam/1194/2004, A(H6N1)/Taiwan/2/2013, A(H7N9)/Anhui/1/2013, or A(H9N2)/Hong Kong/1073/99 could be detected among the study subjects by either HAI or MN assay.

10.1128/mBio.01408-18.4 TABLE S1  Donor information. Download TABLE S1, DOCX file, 0.02 MB. Copyright © 2018 Zhao et al. 2018 Zhao et al. This content is distributed under the terms of the Creative Commons Attribution 4.0 International license.

Using the crystal structures of the major humoral antigen HA, we investigated the humoral antigenic variations of different AIVs compared to A(H1N1)/California/04/2009 (Fig. 1C). Humoral immunogenic sites on the HA protein of influenza A virus are mainly distributed over five conformational sites (Fig. 1D), including four humoral antigenic sites (Sa, Sb, Ca, and Cb) on the HA “head” and a subdominant B-cell epitope on the HA “stem.” Structure-based conservancy analysis of these epitopes revealed that the four antigenic sites on the HA head of the four AIVs A(H5N1)/Vietnam/1194/2004, A(H6N1)/Taiwan/2/2013, A(H7N9)/Anhui/1/2013, and A(H9N2)/Hong Kong/1073/99 were distinct from those of A(H1N1)/California/04. The stem-derived epitope in the HAs of each AIV was comparatively conserved but still contained specific substitutions.

To expand our discovery to other AIV strains, we performed phylogenetic analyses based on HA sequences from different human-infecting H5N1, H6N1, H7N9, and H9N2 strains (Fig. 1E). Different clusters of AIV HAs were generated. Though H1 and H5 are adjacent to each other in phylogeny, there was no cross-reactive serological reactivity to H5N1 among the putative pH1N1-infected population. Thus, the results are consistent with respect to comparisons between the divergent B cell epitopes on the humoral HA antigens of different AIVs and the phenomenon that no cross-reactive antibody titers were observed between the tested AIVs and pH1N1.

### Biased T-cell cross-reactivities.

**FIG 2:**
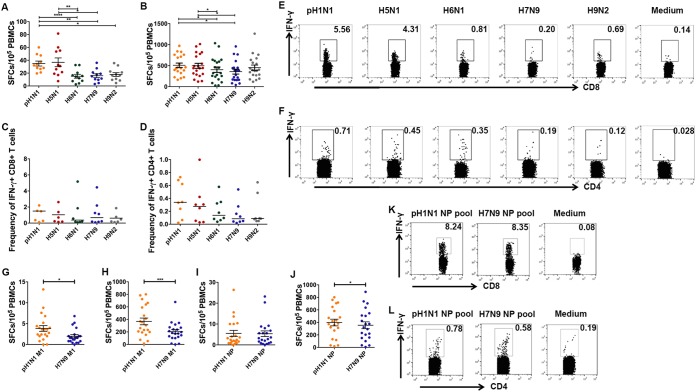
Cross-reactive cellular immune responses to avian influenza viruses. (A) Cellular immune responses of pH1N1 antibody-positive healthy donors to pH1N1, H5N1, H6N1, H7N9, and H9N2. T-cell responses were investigated using freshly isolated PBMCs from individuals (*n* = 11) through IFN-γ ELISPOT assays with live viruses. (B) pH1N1-specific T cells in PBMCs from individuals (*n* = 20) were expanded by stimulation with the pH1N1 M1 peptide pool for 9 days, and the T-cell responses were determined through IFN-γ ELISPOT assays using pools of overlapping peptides derived from influenza virus M1 protein. (C and D) The ratios of influenza virus-specific IFN-γ-secreting cells in CD8^+^ (C) and CD4^+^ (D) T-cell levels were determined using ICS and flow cytometry. The frequencies of virus-specific T-cell responses to AIVs were compared with the frequencies of responses to the pH1N1 M1 pool (*n* = 8). (E and F) Representatives of virus-specific T cells in CD8^+^ (E) and CD4^+^ (F) T cells are shown. (G to J) T-cell responses to the M1 antigen and NP antigen of pH1N1 and H7N9 before and after culturing with M1 pools or NP pools of pH1N1 and H7N9 were compared through IFN-γ ELISPOT and ICS assays. (I) The T-cell responses of freshly isolated PBMCs from the healthy donors to the pH1N1 NP pool and H7N9 NP pool were compared using IFN-γ ELISPOT assays. (J) PBMCs were cultured with the pH1N1 NP pool and tested on the ninth day using IFN-γ ELISPOT assays. The T-cell responses to M1 pools of these two viruses were tested at the same time as the control (G, fresh PBMCs; H, PBMCs cultured *in vitro* with M1). (K and L) NP-specific T-cell responses to pH1N1 and H7N9 tested in ICS assays using PBMCs cultured *in vitro* with the pH1N1 NP pool for 9 days. Data in panels A, B, and G to J are shown as means + SEM (standard errors of the means), and data in panels C and D are shown as medians. The differences among multiple groups were compared using ANOVA (A to D), and differences between two groups were compared using Student’s *t* test (E to H). *, *P* < 0.05; **, *P* < 0.001; ***, *P* < 0.0001.

To investigate potential preexisting T-cell responses to different AIVs among the pH1N1-infected subjects, we stimulated freshly isolated PBMCs from the pH1N1 Ab^+^ cohort via an enzyme-linked immunosorbent spot (ELISPOT) assay with live influenza viruses as stimulators. We found that certain levels of cross-reactive T-cell responses against H5N1, H6N1, H7N9, and H9N2 viruses were detected. Cross-reactive T-cell responses against H5N1 (36.8 spot-forming cells [SFCs]/10^5^ PBMCs) were found at levels similar to those determined for pH1N1-specific T-cell responses (34.9 SFCs/10^5^ PBMCs), but the levels for both were higher than those seen with other AIVs, i.e., H6N1 (15.1 SFCs/10^5^ PBMCs), H7N9 (16 SFCs/10^5^ PBMCs), and H9N2 (17.7 SFCs/10^5^ PBMCs) (Fig. 2A). Lee et al. synthesized the overlapping peptides covering all proteins and found that M1 and NP possess the major immunogenic sites (20). We evaluated cross-reactive T-cell responses to M1 proteins from different AIVs among the pH1N1 M1-cultured PBMCs for 9 days. Though cross-T-cell reactivity was observed for all four tested AIVs, the cross-reactivity to M1 of H5N1 (497 SFCs/10^5^ PBMCs) shown by the pH1N1-specific T cells was higher than that to other AIVs (for H6N1, 404 SFCs/10^5^ PBMCs; for H7N9, 359 SFCs/10^5^ PBMCs) (Fig. 2B). In intracellular cytokine staining (ICS) assays, both the cross-CD8^+^ T cells (4.37% gamma interferon positive [IFN-γ^+^] CD8^+^ CD8^+^) and CD4^+^ T cells (0.27% IFN-γ^+^ CD4^+^ CD4^+^) displayed a bias with respect to H5N1; in contrast, H7N9 presented the lowest cross-T-cell reactivity level, with 1.08% IFN-γ-secreting cells in CD8^+^ T cells and 0.14% IFN-γ-secreting cells in CD4^+^ T cells (Fig. 2C to F), while the T-cell responses of pH1N1-antibody negative individuals did not show such differences in statistics (see Fig. S1A in the supplemental material).

10.1128/mBio.01408-18.1 FIG S1  Cross-reactive cellular immune responses in pH1N1-negative individuals and the conformation shift of HLA-A*2402/H7-P25 confirmed by structure data set 2 at a higher resolution. (A) T-cell responses were investigated using freshly isolated PBMCs from individuals (*n* = 10) through IFN-γ ELISPOT assays using M1 peptide pools. pH1N1-specific T cells in PBMCs were expanded by stimulation with the pH1N1 M1 peptide pool for 9 days, and the T-cell responses were determined through IFN-γ ELISPOT assays using pools of overlapping peptides derived from influenza virus M1 protein. Data are shown as means + SEM (standard errors of the means). Statistical analyses were performed with ANOVA. (B) The conformational superposition of H1-P25 (orange) and H7-P25 (blue; based on data set 2 of HLA-A*2402/H7-P25 at a resolution of 2.3 Å) presented by HLA-A*2402. The residues that have no clear electron density for H7-P25 in HLA-A*2402/H7-P25 are displayed as a dashed line, and the corresponding names of the residues are shown as letters in squares. Download FIG S1, PDF file, 0.1 MB. Copyright © 2018 Zhao et al. 2018 Zhao et al. This content is distributed under the terms of the Creative Commons Attribution 4.0 International license.

### M1 is the dominant contributor to biased T-cell reactivities.

The cross-reactivity to H7N9 was significantly lower than that to other AIVs among the pH1N1 antibody-positive (Ab^+^) subjects. Thus, to investigate the contribution of different cellular antigens of influenza viruses to biased cross-T-cell reactivities, we compared T-cell responses to H7N9 and pH1N1 among the members of the healthy population using overlapping peptides covering the M1 and NP proteins. We found that T-cell reactivity to H7N9 NP was of the same level as the T-cell reactivity to pH1N1 NP as detected in the PBMCs *ex vivo* by ELISPOT assay (Fig. 2I). Subsequently, we established H7N9- and pH1N1-specific T-cell lines *in vitro* by stimulating the PBMCs with an NP peptide pool for 9 days. The responses to NPs of either H7N9 (354 SFCs/10^5^ PBMCs) or pH1N1 (398 SFCs/10^5^ PBMCs) of the T-cell lines remained at the same level (Fig. 2J). In ICS assays, the results seen with IFN-γ-secreting cells in both CD8^+^ and CD4^+^ T cells were similar under conditions of stimulation of H7N9 NP (4.49% IFN-γ^+^/CD8^+^) and pH1N1 NP (4.34% IFN-γ^+^/CD4^+^) (Fig. 2K and L). In contrast, the cross-reactivity to H7N9 M1 (203 SFCs/10^5^ PBMCs) was significantly lower than the reactivity to pH1N1 M1 (368 SFCs/10^5^ PBMCs) itself (Fig. 2G and H).

Subsequently, six immunogenic individual peptides from the pH1N1 M1 pool were identified as responsible for the M1-specific T-cell responses among the subjects via a matrix assay. A biased responsive magnitude against the corresponding peptides from different AIVs was observed, consistent with the diverse substitutions in these peptides (see Fig. S2). Further analysis indicated that these peptides contained previously identified HLA class I- or class II-restricted epitopes (see Fig. S2D and H) and that the immunogenicity can be influenced by the substitutions in different AIVs.

10.1128/mBio.01408-18.2 FIG S2  Biased T-cell cross-reactivities revealed by immunogenic peptides. Six individual peptides (P17, P18, P22, P23, P24, and P33) that led to immunogenicity changes were determined through IFN-γ ELISPOT assays (A to C and E to G). The corresponding sequences of each strain are shown in the tables in the panels below (D and H), and T-cell epitopes identified previously within the long peptides are marked in red letters. The dashes represent residues that are identical to those in the A(H1N1)/California/04/2009 virus, while residues in other strains that differ from those in the A(H1N1)/California/04/2009 are shown in letters. Download FIG S2, PDF file, 0.3 MB. Copyright © 2018 Zhao et al. 2018 Zhao et al. This content is distributed under the terms of the Creative Commons Attribution 4.0 International license.

### Phenotypes of the cross-reactive T-cell.

**FIG 3:**
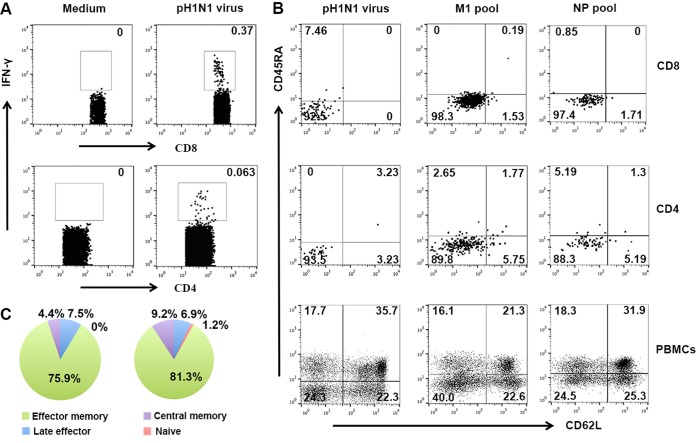
Phenotype and functional characterization of influenza virus-specific T cells. (A) Frequencies of influenza virus-specific T cells were determined via IFN-γ staining using ICS assays. (B) The memory phenotype of influenza virus-specific T cells was determined by the use of CD62L and CD45RA after stimulation with pH1N1 live virus, the M1 peptide pool, and the NP peptide pool of pH1N1. Characteristics of the virus-specific CD8^+^ T cells are shown on the first row in panel B, and those of virus-specific CD4^+^ T cells are shown on the second row in panel B. The cross-quadrant gate on the virus-specific T cells was determined by the gate of the PBMCs, shown on the third row in panel B. (C and D) The median ratios of each phenotype of the IFN-γ-secreting CD8^+^ T cells (C) and CD4^+^ T cells (D) from six individuals are shown in the pie graphs.

To further investigate the functional subset of the cross-reactive T cells, we detected the memory phenotypes of pH1N1-specific IFN-γ^+^ T cells. Memory phenotypes of the IFN-γ-secreting cells with respect to all of the different antigens, including live pH1N1 virus and the M1 pool and NP pool of pH1N1, were determined by analysis of the CD45RA and CD62L data (Fig. 3A and B). The results showed that for all the antigens, the effector memory (CD45RA^−^ CD62L^−^) T-cell subset dominated the IFN-γ-secreting cells both among CD8^+^ and CD4^+^ T cells. For instance, 75.9% (median) of the IFN-γ^+^ CD8^+^ T cells and 81.3% (median) of the IFN-γ^+^ CD4^+^ T cells specific for M1 presented an effector memory phenotype (Fig. 3C).

### Correlation of the internal proteins and biased T-cell cross-reactivities.

**FIG 4:**
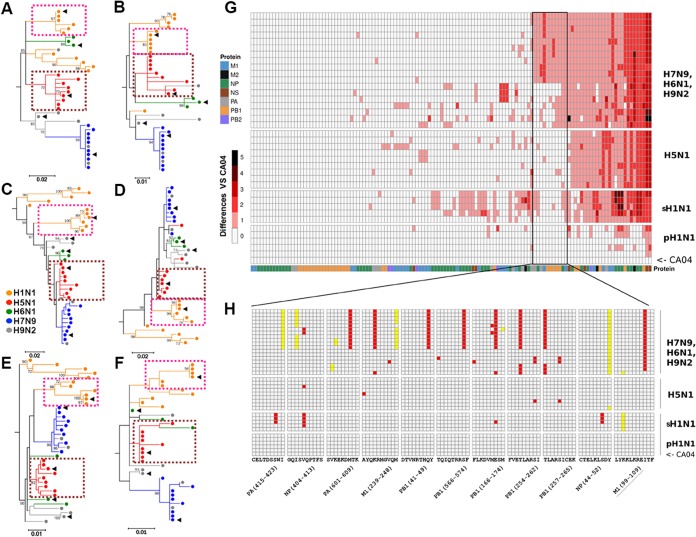
Immunoinformatic analysis of cellular antigenicities. Phylogenetic analyses based on protein sequence (A, C, and E) and CTL epitopes (B, D, and F) of the T-cell antigens M1, NP, and PB1 were performed using MEGA6. The human-infecting AIVs (H5N1, H6N1, H7N9, and H9N2) together with the H1N1 strains (including pH1N1 strains and seasonal H1N1 strains from before 2009 and also 1918 H1N1) are represented with different colors. The clusters of pH1N1 strains are denoted with purple dotted squares. The clusters of human-infecting H5N1 strains are presented as brown dotted squares. The black triangles indicate the five virus strains A (H1N1)/California/4/2009, A(H5N1)/Vietnam/1194/2004, A(H6N1)/Taiwan/2/2013, A(H7N9)/Anhui/1/2013, and A(H9N2)/Hong Kong/1073/99 used in this study. (G) Bioinformatics analysis of the potential cellular antigenicities of different AIVs based on the previously determined T-cell epitopes available in the Immune Epitope Data Base (IEDB) on the heat map. Peptides within different viruses were extracted as predicted T-cell epitopes of the representative sequences. We counted the number of residues of each predicted T-cell epitope using A/California/04/2009 as a reference. The maximum-likelihood phylogenetic trees for T-cell epitope sequences were constructed using Molecular Evolutionary Genetics Analysis MEGA6 software with the JTT model and 1,000 bootstrap replicates. The virus and peptide information used for analysis of the data presented in panel G is presented in Table S6 and S7. Eleven epitopes that are conserved in H5N1 and pH1N1 strains but that different from those in other AIVs are framed with a black rectangle. (H) The sequences of each strain were compared to the sequence of A(H1N1)/California/04/2009 virus, and mutant sites are highlighted in red (potential TCR docking sites) or yellow (anchor residue sites). Peptide M1 (99–109; underlined) indicates peptide H1-P25. The sequence information from the 11 peptides is presented in Table S3. pH1N1, 2009 pandemic influenza virus cluster; sH1N1, seasonal H1N1 strains before 2009 (but the data also include the 1918 H1N1 strain).

T-cell immunity plays an important role in protection from heterosubtypic influenza infections when antibodies do not work well. To better investigate the correlation of the internal proteins and T-cell cross-reactivities, we performed phylogenetic analyses of the predominant influenza virus T-cell immunogens, i.e., M1, NP, and PB1, based on the full-length protein sequences (Fig. 4A and C and E). We found that the HA relationship of the AIVs did not reflect the biased cross-T-cell reactivities. The M1 protein from different H5N1 strains and that from pH1N1 were not very clearly closely related to each other, and neither were NP and PB1. Cross-reactive T cells targeted different CD8^+^ or CD4^+^ T-cell epitopes of AIVs covering special sequences of the antigens. Thus, we hypothesized that the phylogenetic relationship in terms of the T-cell epitopes from different AIVs may correspond to the biased cross-T-cell reactivities. We retrieved the previously defined HLA class I-restricted peptides located within the internal M1, NP, and PB1 proteins of pH1N1 and the corresponding peptides within different AIVs and plotted their phylogenetic relationship (Fig. 4B and D and F). The T-cell epitope evolution of pH1N1 is close to that of H5N1 but distant from those of H7N9, H9N2, and H6N1, which is consistent with the biased cross-T-cell reactivity to H5N1 but not to the other strains.

To trace the full view of CD8^+^ immunogenic features of pH1N1 and AIVs, we performed bioinformatic analyses of potential cellular antigenicity for different viruses based on previously determined T-cell epitopes available from the Immune Epitope Data Base (IEDB). We analyzed 38 representative human-infecting AIV strains as well as the recent seasonal H1N1 stains. A total of 266 CTL epitopes were retrieved from IEDB and then mapped to each strain (up to 30 December 2016). We filtered out 129 internal protein epitopes to perform further analysis (see Table S2). Clustering analysis indicated that the H1N1 stains are located in one cluster (see Fig. S3A). For different human-infecting AIV strains, H5N1 strains remained close to pH1N1 compared to the distance of other AIVs (i.e., H7N9, H6N1, H9N2) to pH1N1 (Fig. 4G). The H7N9 and H9N2 strains were intermixed with each other, which may have been related to the shared internal genes of these AIV subtypes in China (5). Seasonal H1N1 influenza viruses before 2009, such as A(H1N1)/Brisbane/59/07, were located in a cluster that was adjacent to but distinct from that of pH1N1, indicating a cellular antigenic transition of pH1N1 compared to previous H1N1 stains.

10.1128/mBio.01408-18.3 FIG S3  Clustering analysis of H1N1 stains and human-infecting avian influenza viruses. (A) Clustering analysis of H1N1 stains and human-infecting avian influenza viruses with human epitopes. A total of 266 CTL epitopes in IEDB (http://www.iedb.org/) were retrieved and then mapped to each strain (up to 30 December 2016). These epitopes were mapped to the proteins of A/California/04/2009. Protein sequences of representative strains for H1N1, H5N1, H6N1, H7N9, and H9N2 were downloaded from the GISAID EPIFLU database (http://platform.gisaid.org/epi3/frontend), and peptides with the sequences were extracted as predicted T-cell epitopes of the representative sequences. A/California/04/2009 was used as a reference. The maximum-likelihood phylogenetic trees of T-cell epitope sequences were constructed using Molecular Evolutionary Genetics Analysis MEGA6 software. Different subtypes of influenza viruses are denoted with different colors. The black triangles indicate the five virus strains A (H1N1)/California/4/2009, A(H5N1)/Vietnam/1194/2004, A(H6N1)/Taiwan/2/2013, A(H7N9)/Anhui/1/2013, and A(H9N2)/Hong Kong/1073/99 used in this study. (B) Maximum-likelihood tree of joint sequences of 122 mouse epitopes. Bootstrap values of over 70% are indicated on branches. Strains of H1N1, H5N1, H6N1, H7N9, and H9N2 are colored as orange, red, green, blue, and gray, respectively. The scale bar under the tree represents number of substitutions per site. (C) Comparison of the 122 mouse epitopes in the 38 representative strains. The columns represent epitopes, and the rows represent strains. The color of each cell represents the number of different residues of each epitope compared with those of A(H1N1)/California/4/2009.The strains are grouped by subtypes, and the order of groups corresponds to the cluster order of the maximum-likelihood tree of joint sequences of 122 mouse epitopes. Download FIG S3, PDF file, 0.2 MB. Copyright © 2018 Zhao et al. 2018 Zhao et al. This content is distributed under the terms of the Creative Commons Attribution 4.0 International license.

10.1128/mBio.01408-18.5 TABLE S2  Human epitopes in use. Download TABLE S2, DOCX file, 0.1 MB. Copyright © 2018 Zhao et al. 2018 Zhao et al. This content is distributed under the terms of the Creative Commons Attribution 4.0 International license.

Comparing the different variations of the T-cell epitopes between pH1N1 and various AIVs, a cluster that included 11 T-cell epitopes was found to be conserved in H5N1 and pH1N1 strains but presented different variations in other AIVs, either in major histocompatibility complex class I (MHC-I)-anchoring or T-cell receptor (TCR)-docking positions (30) (Fig. 4G and H; see Table S3). This cluster of epitopes may have a key role in determining the bias of cross-T-cell reactivities to different AIVs in the healthy population.

10.1128/mBio.01408-18.6 TABLE S3  Eleven key epitopes showed conservation in H1 and H5 subtypes. Download TABLE S3, DOCX file, 0.1 MB. Copyright © 2018 Zhao et al. 2018 Zhao et al. This content is distributed under the terms of the Creative Commons Attribution 4.0 International license.

We also performed bioinformatic analyses using influenza virus-derived T-cell epitopes with restrictions of different mouse MHC alleles (H-2D^b^, H-2K^b^). Both the clustering and phylogenetic analyses (see Fig. S3B and C) showed that the mouse epitope-based H1N1 lineage was still adjacent to H5N1 but far from other AIVs, including H7N9.

### Molecular basis of the biased T-cell cross-reactivities.

**FIG 5:**
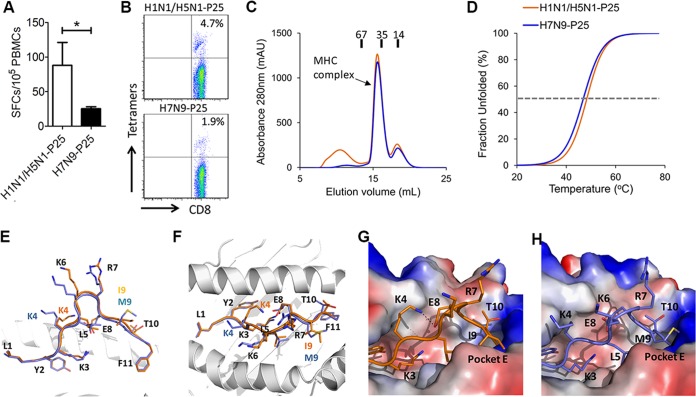
Molecular bases of the biased cross-T-cell reactivities. (A) An individual CD8^+^ T-cell epitope that has antigenic variation between pH1N1 (H1-P25, similar peptide in H5N1) and H7N9 (H7-P25) was determined among different donors with HLA-A*2402 restrictions. PBMCs from different donors were tested via the IFN-γ ELISPOT assay by stimulation with individual H1-P25 and H7-P25 peptides. Abbreviation: SFC, spot-forming cell. (B) Tetramers of HLA-A*2402 complexed to H1-P25 or H7-P25 were prepared and utilized to stain influenza virus-specific T cells. (C) Binding of peptides H1-P25 and H7-P25 to HLA-A*2402 was elucidated by *in vitro* refolding. Peptides with the ability to bind to HLA-A*2402 help the HLA-A*2402 H chain to refold *in vitro* in the presence of β_2_m. After properly refolding, the high absorbance peaks of HLA-A*2402 with the expected molecular mass of 45-kDa eluted at the estimated volume of 16 ml on a Superdex 200 10/300 GL column. The profile is marked with the approximate positions of the molecular mass standards of 67.0, 35.0, and 14.0 kDa. (D) Thermostability of HLA-A*2402/H1-P25 and HLA-A*2402/H7-P25 complexes as revealed by CD spectroscopy. The *T*_*m*_s of different peptides are indicated by the gray dashed line at the 50% unfolded fraction. (E) The conformational superposition of H1-P25 (orange) and H7-P25 (blue; based on data set 1 of HLA-A*2402/H7-P25) presented by HLA-A*2402 observed by a side view. The residues are denoted with a single-letter name together with a position number. The I9M substitution and changed-conformation residue K4 of H1-P25 (orange) and H7-P25 (blue; based on data set 1 of HLA-A*2402/H7-P25) are labeled with different colors. (F) The conformational superposition of H1-P25 (orange) and H7-P25 (blue) presented by HLA-A*2402 through a top view. (G) The binding of peptide H1-P25 to HLA-A*2402. Residue I9 inserts its side chain into hydrophobic pocket E. Residue K4 forms a hydrogen bond with E8. The peptide binding groove is shown by the vacuum electrostatic surface potential of HLA-A*2402 H chain. (H) The location of peptide H7-P25 within the HLA-A*2402 groove based on data set 1 of HLA-A*2402/H7-P25. The side chain of the substituted residue M9 protrudes from pocket E. Residue K4 is also solvent exposed and is ready for TCR docking.

As mentioned above, there was a region covering 11 nonconserved short peptides which was highlighted in the heat map (Fig. 4H). The 11 epitopes had a common feature: the amino acids which showed substitutions at the anchoring positions and/or at the exposed positions were substituted frequently (see Table S3). This indicates that the variable antigenicity of the nonconserved peptides may be contributed by substitution-dependent intervention of MHC binding and/or TCR recognition. In this study, more than half (54.5%) of the subjects had the HLA-A*2402 allele, and M1 protein-derived peptide H1-P25 (M1 99–109; LYKKLKREITF in pH1N1) with HLA-A*2402 restriction is 1 of the 11 peptides. P25 is conserved between H1N1 and H5N1 (named peptide H1-P25) but has a dominant mutation with substitutions at position 9 from Ile to Met (I9M) (named peptide H7-P25) in H7N9 and H9N2 and Ile to Val (I9V) in H6N1. In HLA-A*2402^+^ individuals, we confirmed that peptide H7-P25 could induce lower cross-reactivity than peptide H1-P25 only in pH1N1-specific T cells (Fig. 5A).

To characterize the binding of peptide H1-P25 derived from pH1N1 (or H5N1) and H7/H9 variant H7-P25 to HLA-A*2402, *in vitro* renaturing (Fig. 5B) and circular dichroism (31) assays of the HLA-A*2402/peptide complexes were performed (Fig. 5C). The refolding efficiencies of both the HLA-A*2402/H1-P25 and HLA-A*2402/H7-P25 complexes were high (Fig. 5B). Further, the HLA-A*2402/H1-P25 complex was more thermally stable (with a melting temperature [*T*_*m*_] of 48.8°C) than the HLA-A*2402/H7-P25 complex (with *T*_*m*_ = 46.4°C) (Fig. 5C). Despite the fact that the I9M mutation is not located in the traditional primary anchoring positions of HLA I binding peptides, peptide H7-P25 displayed a minor decreased binding affinity for HLA-A*2402 compared to H1/H5-derived peptide H1-P25.

To further interpret the immunogenicity transition at the molecular level, we determined the crystal structures of HLA-A*2402 in complex with peptide H1-P25 or H7-P25 (data set 1) (see Table S4). The overall conformations of main chains were similar in the two structures (Fig. 5D and E). However, in the HLA-A*2402/H1-P25 structure, residue Ile9 inserts inside the E pocket of the HLA-A*2402 groove (Fig. 5F). In contrast, in the HLA-A*2402/H7-25 complex, mutated residue Met9 is too large to be accommodated in the E pocket of HLA-A*2402 and, thus, its side chain protrudes from the peptide binding groove and might be contacted by the TCR (Fig. 5G). Another structural variation is contributed by Lys4. Although this residue is conserved between the P25 peptides of pH1N1 and H7N9, the salt bridge formed between Lys4 and Glu8 in HLA-A*2402/H1-P25 is not present in the structure of HLA-A*2402/H7-P25. Without the constraint of the salt bridge, Lys4 of peptide H7-P25 in HLA-A*2402/H7-P25 is exposed to solvent instead of being partially buried in the C pocket as in the HLA-A*2402/H1-P25 structure. Due to the low resolution (3.3 Å) of the first data set from HLA-A*2402/H7-P25, we collected another data set which had higher resolution (2.3 Å) (see Table S4). Though no clear electron density for residues in the middle portion of H7-25 was observable on the basis of data set 2 of HLA-A*2402/H7-P25, the conformations of residues Lys4 and Met9 from H7-P25 in data set 2 were still different from those from H1-P25 but were similar to those from H7-P25 in data set 1 (see Fig. S1B).

10.1128/mBio.01408-18.7 TABLE S4  Data collection and refinement statistics. Download TABLE S4, DOCX file, 0.02 MB. Copyright © 2018 Zhao et al. 2018 Zhao et al. This content is distributed under the terms of the Creative Commons Attribution 4.0 International license.

Taking these data together, though the H7-P25 peptide has only one dominant I9M mutation, this mutation influences the antigenicity of the peptide, most likely by altering both HLA binding and TCR recognition. This may illustrate a common mode of antigenic variation of the 11 nonconserved peptides between the H1N1/H5N1 cluster and other AIVs (H6N1, H7N9, and H9N2) that induce a biased scale of cross-reactive T-cell responses.

### Biased T-cell cross-reactivities provided distinct cross-protection efficacies against AIVs.

**FIG 6:**
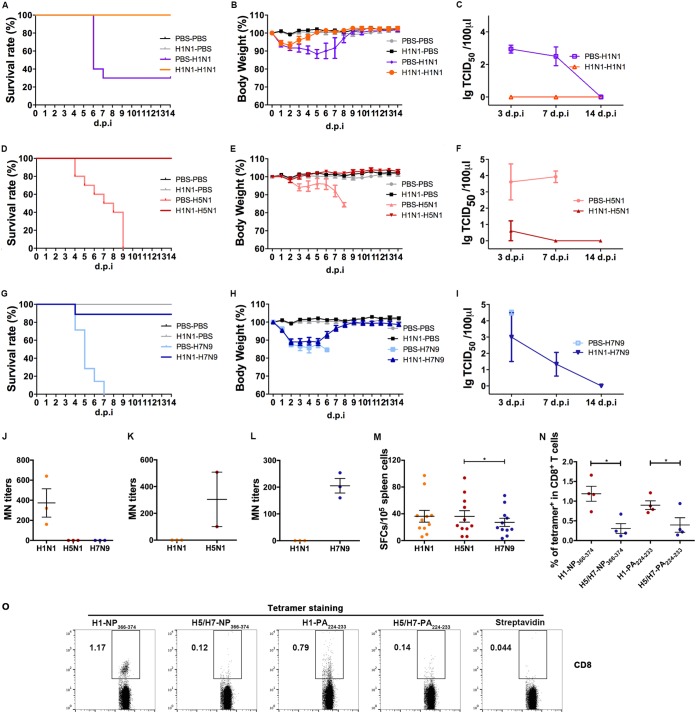
Different cross-protection efficacies against H5N1 and H7N9 provided by pH1N1-specific T cells. Mice were preinfected with pH1N1 virus (10^3.8^ TCID_50_) and, 28 days later, challenged with a lethal dose of A(H1N1)/California/4/2009 (10^5.8^ TCID_50_) (A to C), A(H5N1)/Vietnam/1194/2004 (10^5.2^ TCID_50_) (D to F), or A/Anhui/1/2013 (H7N9) (10^5.6^ TCID_50_) (G to I), as well as with PBS as a control. Survival status, body weight, and viral titration of lung homogenates were determined (A to I). The numbers of mice used for survival and weight analyses were as follows: *n* = 9 for PBS-PBS, *n* = 10 for PBS-H1N1, *n* = 10 for PBS-H5N1, *n* = 7 for PBS-H7N9, *n* = 10 for H1N1-PBS, *n* = 10 for H1N1-H1N1, *n* = 10 for H1N1-H5N1, and *n* = 9 for H1N1 to H7N9. (A, D, and G) Survival was recorded following the second virus infection. (B, E, and H) Body weight losses are presented as mean percentages of the weight differences of the animals relative to their weight on the day prior to inoculation. (C, F, and I) Lung homogenate samples were collected at 3, 7, and 14 days postinfection. Data are shown with the mean ± SEM virus titers of three mice per time point. (J) Levels of antibodies to pH1N1, H5N1, and H7N9 of H1N1-positive serum samples were tested by MN assay. (K and L) Levels of antibodies to pH1N1 and H5N1 in H5N1-positive serum samples (K) and levels of antibodies to pH1N1 and H7N9 of H7N9-positive serum samples (L) were also tested. (M) Cross-reactive T-cell responses to H5N1 and H7N9 of spleen cells isolated from mice infected with pH1N1 for 2 weeks were determined by IFN-γ ELISPOT assays. (N and O) pH1N1-specific CD8^+^ T cells of pH1N1-primed mice were stained with tetramers H1-NP_366−374_ (complex between H-2D^b^ and peptide from H1N1), H5/H7-NP_366−374_ (complex between H-2Db and peptide from H5N1/H7N9), H1-PA_224−233_ (complex between H-2D^b^ and peptide from H1N1), and H1-PA_224−233_ (complex between H-2D^b^ and peptide from H5N1/H7N9). CD8^+^ T cells were subsequently selected for the analysis of tetramers (*n* = 4). Differences between two groups were compared using Student’s *t* test (J to L and N), and differences among multiple groups were compared using ANOVA (M). *, *P* < 0.05.

Considering the unequal cross-reactivities to different AIVs exhibited by pH1N1-specific T cells, especially with respect to the difference between H5N1 and H7N9, the next issue that we addressed was whether these T-cell immunogenicity variations can lead to different heterosubtypic protections against these AIVs. We used pH1N1 virus at sublethal doses to prime mice, and 28 days later, the mice were intranasally infected with lethal doses of pH1N1, H5N1, or H7N9. For homologous challenge of pH1N1, the protection ratio was 100% (Fig. 6A). The rate of heterosubtypic protection against H5N1 challenge was also 100% (Fig. 6D), but the rate of protection against H7N9 challenge was 89% (Fig. 6G). The mice in the homologous pH1N1 challenge group (Fig. 6B) and the pH1N1 primed-H5N1 challenge group (Fig. 6E) displayed no or minor body weight losses, while the mice in the pH1N1 primed-H7N9 challenge group (Fig. 6H) had a body weight loss of >10% in the first 7 days after H7N9 challenge. Concerning virus shedding in the lungs after infection, no virus was detected by 3 days postinfection (d.p.i.) or afterward for the homologous challenge of pH1N1 (Fig. 6C). As for the pH1N1-primed H5N1 group (Fig. 6F), the H5N1 virus load was much lower than that seen with the unprimed group by 3 dpi and disappeared by 7 dpi. In the H7N9 groups (Fig. 6I), the primed group had a detectable virus load by 3 and 7 dpi.

To distinguish the contributions of neutralizing antibodies and T-cell responses in the heterosubtypic protection against AIVs, we performed MN and T-cell response detection assays in mice infected with different influenza viruses at sublethal dosages. In the pH1N1-infected group, pH1N1-specific neutralizing antibodies were detectable on day 14, and no cross-neutralization antibody titers to H5N1 or H7N9 could be detected (Fig. 6J) and similarly, there was no cross-neutralization antibody titers to pH1N1 detected in serum of the H5N1- or H7N9-infected group (Fig. 6K and L). The pH1N1-specific T-cell response in the mice was found to be 363 SFCs/10^6^ splenocytes at 14 dpi with pH1N1 (Fig. 6M). Meanwhile, cross-reactive T-cell responses to either H5N1 or H7N9 were found in the pH1N1-infected mice. However, the cross-reactive T-cell responses to H7N9 (273 SFCs/10^6^ splenocytes) were lower than those against H5N1 (361 SFCs/10^6^ splenocytes). These results indicated that bias of cross-reactive T-cell responses induced by pH1N1 may lead to different protection efficacies against H5N1 and H7N9. We also prepared the tetramers of two immunodominant H-2D^b^-restricted T-cell epitopes, NP_366–374_ and PA_224−233_, and their H5N1/H7N9 mutants. The tetramer staining of the splenocytes from the mice infected by H1N1 for 28 days showed that the mice possessed robust H1-NP_366−374_ and H1-PA_224−233_ peptide-specific CD8^+^ T cells (1.2% and 0.9%), whereas certain T cells cross-recognizing the H5N1/H7N9 mutants could also be detected (0.31% and 0.40%) (Fig. 6N and O). However, the ratios of mutant peptides H5/H7-NP_366−374_ and H1/H7-PA_224−233_ were lower, which may still have contributed to the cross-protection.

## DISCUSSION

Here, we found that pH1N1-specific T cells had biased cross-reactivities to different human-infecting AIVs, while no preexisting neutralizing antibodies were detected. The cross-cellular immune response to H5N1 in previously pH1N1-infected subjects was higher than those to H6N1, H7N9, and H9N2, correlating with heterosubtypic protection in an animal model. Epitope-based phylogenetic analysis demonstrated that the H5N1 subtype possesses a cluster of conserved epitopes with pH1N1 that may lead to the observed cross-antigenicity. Peptide-MHC (pMHC) structure determination indicated a molecular basis for the immune cross-reactivity between pH1N1 and H5N1, as well as the lack of cross-reactivities toward other AIVs, especially H7N9.

Antibody-mediated neutralization is the direct inhibition of viral infection (32). The elicitation of a neutralizing-antibody response is a correlate of protection for vaccines and contributes to protection against many viral infections (33). In particular, HA imprinting in childhood could provide lifelong protection against severe infection and death from emergent viruses (34). However, a previous study showed that no H7N9-specific antibody titers were detected in the 1,544 serum samples collected before the emergence of H7N9 from poultry workers, most of whom were born after 1968, when H3N2 emerged (18). This indicated that cross-reactive serological immunity against H7N9 virus did not preexist among healthy young populations. In our study, the levels of neutralizing antibodies that cross-recognized H5N1 and H7N9 were also undetectable, which may indicate a critical role of T cells in heterosubtypic protection. Recent studies indicated that HA-specific CD4^+^ T cells do not possess a dominant role compared to the internal proteins such as M1 and NP (20, 35). Therefore, HA-specific immunity may not be sufficient to explain the cross-protection against AIV by the immunity to previous seasonal influenza viruses. According to our bioinformatics analyses, the heat map of all available T-cell epitopes among human-infecting viruses indicated that the accumulation of varied epitopes may hinder cross-T-cell reactivities. In particular, a cluster of conserved T-cell epitopes shared by H5N1 and H1N1 may be related to cross-protection against H5N1. These epitopes could be considered for use in the development of vaccines preventing H1N1 and H5N1 infections. In contrast, T-cell recognition of the mutant viral epitopes in H7N9 was significantly decreased due to the poor T-cell activation threshold and disrupted peptide-HLA interactions. Although low cross-reactivity of the variable peptides may also exist due to TCR conformational plasticity, their protective effect remains to be determined (36). We also detected a certain level of cross-reactive T-cell responses against AIVs existing among different individuals, which may be contributed by conserved T-cell epitopes. Greenbaum et al. showed that a large fraction of conserved T-cell epitopes in seasonal influenza virus could induce significant T-cell responses; as such preexisting T-cell immunity may decrease the severity of a variant strain infection (37). Our previous work also confirmed a dominant role for conserved T-cell epitopes in anti-influenza virus responses (30).

Although there have been hundreds of influenza virus-specific epitopes identified across proteins using a range of epitope identification techniques, the majority of the conserved epitopes are derived from the internal proteins M1, NP, and PB1 (37–39). Lee and others reported that M1 and NP possess the major immunogenicities among the internal proteins, followed by PB1 and PA (20). They also found that the recognition frequency of M1 protein was higher than those of the other internal proteins. However, the identity of the internal proteins which possess a dominant influence on T-cell cross-reactivity was still unknown. On the basis of previous researches, we compared the T-cell responses against H1N1 and H7N9 among healthy donors with overlapping peptide pools of NP and M1, respectively, in the present study. NP-specific T-cell cross-reactivity against H7N9 showed a high level among the members of the pH1N1 Ab^+^ population. Interestingly, we found biased T-cell cross-reactivities in responses to M1 proteins derived from different AIVs. The similarity of the M1 sequences in the two strains was lower (92%) than those of NP (93%) and other internal proteins (PB1 [96%], PB2 [97%], and PA [96%]). Besides, the influences of the substitutions on immunogenicity would be different among different proteins, which may also contribute to varied cross-reactivity of the internal proteins between H1N1 and H7N9. Mutations in the M1 protein might have a larger influence on its immunogenicity. Considering the immunogenicity and dissimilarity conservation of these two proteins, we proposed that M1 might have a more dominant influence in eliciting T cell immunity among influenza viruses and chose M1 as the stimulus in the experiments that followed that assessed T cell cross-reactivity and heterogeneous protection. Thus, like the humoral response in HA, minor variations among the immunodominant epitopes may impact cross-T-cell reactivities, which should be carefully considered during the development of universal vaccines based on the M1 proteins of influenza viruses.

The activation of T cells could be affected mainly by two factors: the stability of the peptide-MHC (pMHC) complex and the interaction between TCR and pMHC. Minor substitutions of the residues on the middle bulged region of the peptides can abrogate T-cell recognition (40). Also, the thermal stability of pMHC could influence T-cell activation. Peptide Gag_180–188_ derived from HIV could be recognized by HLA-B*07:02, HLA-B*42:01, HLA-B*42:02, and HLA-B*81:01. However, Gag_180–188_ showed poor thermal stability with HLA-B*42:02 and elicited weaker T-cell responses (41). Also, the substitutions of the primary or secondary anchor residues may completely change the antigenicity of the peptides (42). As previously determined in structural studies, mutational escape in T-cell epitopes contributed to the antigenic disaccord (43, 44). Gras et al. investigated the cross-reactive T-cell responses against HLA-B7 supertype-restricted variable epitope NP_418−426_ in humans (43). They found that cross-reactive CD8^+^ T-cell immunity did not exist between the pH1N1 virus and recent seasonal influenza viruses due to structural variation of the solvent-exposed residues in T-cell epitopes that can be recognized by TCRs. Mutations of anchor residues (or partially solvent-exposed secondary anchors) can also dramatically decrease CD8^+^ T-cell responses and result in delayed viral clearance (45). Moreover, the substitutions may also lead to an induced fit on the helices of MHC-I that form the peptide-binding groove (46) and thus impact TCR recognition (47). In the structures of HLA-A*2402 with H1/H5-derived peptide H1-P25 and H7/H9-specific epitope H7-P25, the I9M substitution influenced the solvent-exposed surface and decreased the force of anchoring of the peptide to HLA-A*2402. This indicated that variable antigenicities of nonconserved peptides could be largely influenced by both exposed positions for TCR docking and anchoring positions for MHC binding, which should be investigated in the HLA-A*2402 cohort.

Influenza virus-specific effector memory T cells were shown to be able to efficiently reduce the pulmonary viral titer early during the secondary infection as they accumulated in the lungs with rapid kinetics (48). A previous study reported the correlation of preexisting T cells with memory phenotype to the conserved pH1N1 epitopes in core proteins M1 and NP with clinical outcomes after incident pH1N1 infection (12). We also found that most of the functional cross-reactive T cells were dominated by the effector memory phenotype. In recent years, while hundreds of AIV-infected cases had been reported, a large number of latent AIV infections also existed among LPM workers, as revealed by serological surveillance (49). Our study showed that prior pH1N1 infection led to heterosubtypic protection, and others indicated that the cross-reactive memory T cells were critical in heterosubtypic protection against H7N9 with different hierarchies in mice (50, 51). Determining whether the memory T cells induced by seasonal influenza viruses provide cross-protection against AIV infection in humans requires further investigation.

Overall, our study revealed preexisting but biased T-cell reactivity of pH1N1 influenza virus to human-infecting AIVs which provided distinct protection toward each subtype. This cross-reactive T-cell recognition had a regular pattern depending on the T-cell epitope matrix derived from AIVs and seasonal influenza viruses. Thus, efforts to develop heterosubtypic protection-oriented universal vaccines against influenza viruses should consider the pattern of cross-T-cell immunity.

## MATERIALS AND METHODS

### Ethics statement.

The Ethics Review Committee, National Institute for Viral Disease Control and Prevention, Chinese Center for Disease Control and Prevention (China CDC), approved this study [project approval number IVDC2014(005)]. All of the subjects provided written informed consent for the studies performed on their samples and publication of their cases. The donors were identified anonymously as donor 1 to donor 35. Animals and eggs used in this study (6-to-8-week-old female C57BL/6 mice and 9-to-11-day-old embryonated chicken eggs) were bought from Beijing Vital River Laboratory Animal Technology Co., Ltd. Animal experiments were approved by Animal Experimental Ethics Board, China CDC (approval number 20140225011). The study was conducted in accordance with the principles of the Declaration of Helsinki and the standards of good clinical practice (as defined by the International Conference on Harmonization).

### Subjects and samples.

A total of 35 healthy volunteers were recruited from November 2013 to April 2014. Serum samples were collected from coagulation-promoting tubes (BD Vacutainer) from all donors, and PBMCs were isolated from anticoagulant blood by Ficoll-Paque density centrifugation from 30 of the samples (see Table S1 in the supplemental material). Volunteers were given questionnaires to confirm whether they had received influenza vaccination or had caught cold. None of the subjects had previously received influenza vaccines. And no symptoms of influenza virus infection were reported during the sampling period. HLA class I genotyping of the donors was performed using LABType SSO (One Lambda).

### Viruses.

The A(H1N1)/California/4/2009 virus was propagated in Madin-Darby canine kidney cells (MDCK) (the Cell Bank of Chinese Academy of Sciences). MDCK cells were cultured in Dulbecco’s modified Eagle’s medium (DMEM) (Gibco) containing 10% fetal bovine serum (Gibco). The A(H5N1)/Vietnam/1194/2004, A(H6N1)/Taiwan/2/2013, A(H7N9)/Anhui/1/2013, and A(H9N2) /Hong Kong/1073/99 viruses were propagated in 9-to-11-day-old specific-pathogen-free (SPF) embryonated chicken eggs (Beijing Vital River Laboratory Animal Technology Co., Ltd.).

### Serological assays.

HAI and MN assays were performed using A(H1N1)/California/4/2009, A(H5N1)/Vietnam/1194/2004, A(H6N1)/Taiwan/2/2013, A(H7N9)/Anhui/1/2013, and A(H9N2)/Hong Kong/1073/99 inactive virus antigens, in accordance with WHO protocols (18). Titers of ≥1:40 for HAI and 1:80 for MN were defined as seropositive.

### Design and synthesis of influenza virus-derived peptide pools.

Peptides (15- to 18mers) overlapping by 11 residues and spanning the M1 protein of A(H1N1)/California/4/2009, A(H5N1)/Vietnam/1194/2004, A(H6N1)/Taiwan/2/2013, A(H7N9)/Anhui/1/2013, and A(H9N2)/Hong Kong/1073/99 were designed and synthesized as previously described (52). The peptides with substitutions within these viruses were divided into corresponding pools (see Table S5). Overlapping peptides covering NPs of A(H1N1)/California/4/2009 and A(H7N9)/Anhui/1/2013 were synthesized according to a similar strategy.

10.1128/mBio.01408-18.8 TABLE S5  M1 overlapping peptide pools of influenza viruses. Download TABLE S5, DOCX file, 0.02 MB. Copyright © 2018 Zhao et al. 2018 Zhao et al. This content is distributed under the terms of the Creative Commons Attribution 4.0 International license.

### Expansion of influenza virus-specific T-cell lines *in vitro.*

PBMCs from donors were incubated with influenza virus peptide pools in RPMI 1640 (Gibco) containing 10% fetal bovine serum (HyClone) at 37°C with 5% CO_2_ at a density of 2.5 × 10^6^ cells/ml in a 24-well plate. On day 3, 20 U/ml recombinant human IL-2 (rhIL-2) was added to the medium (53). Half of the medium was changed on day 7 with supplementation by rhIL-2. Cells were harvested and tested for the presence of influenza virus-specific T cells on day 9.

### ELISPOT assay.

Antigen-specific T lymphocyte responses were detected via an IFN-γ-secreting ELISPOT assay (BD). Briefly, 96-well ELISPOT plate membranes were coated with anti-human IFN-γ antibody at 4°C overnight before use. PBMCs from donors were incubated in wells (2.5 × 10^5^/well *ex vivo* for freshly isolated PBMCs and 5 × 10^4^/well for *in vitro*-cultured T-cell lines) along with viruses (multiplicity of infection [MOI] of 3) or peptide pools (2 µM for individual peptides) for 18 h at 37°C with 5% CO_2_, as well as with phytohemagglutinin (PHA) as a positive control for nonspecific stimulation. Cells incubated without stimulation were employed as a negative control. After incubation, cells were removed and, in turn, plates were processed with biotinylated IFN-γ detection antibodies, streptavidin-horseradish peroxidase (HRP) conjugate, and substrate. The development of the spots was stopped by thoroughly rinsing with water. The numbers of the spots were captured and quantified with an automatic ELISPOT reader and image analysis software (Cellular Technology Limited). The threshold of the positive responses set for *ex vivo* ELISPOT was ≥20 SFCs/10^6^ PBMCs.

### Intracellular cytokine staining and flow cytometry.

After pH1N1-specific T cells were expanded *in vitro* for 9 days, T-cell lines were washed and rested for 2 h. These cells were then stimulated with a specific peptide pool for 2 h and incubated with GolgiStop/monensin (BD Bioscience) for an additional 4 h at 37°C in 5% CO_2_. Then, cells were harvested and stained with a panel of surface MAbs in fluorescence-activated cell sorter (FACS) buffer (0.5% bovine serum albumin) for 30 min on ice, including fluorescein isothiocyanate (FITC)-anti-CD8 (BD Pharmingen 555366), peridinin chlorophyll protein (PerCP)-anti-CD4 (BioLegend 317432, clone OKT4), phycoerythrin (PE)-anti-CD62L (BD Pharmingen 555544), and V450-anti-CD45RA (BD Biosciences 560362). Subsequently, cells were fixed with BD fix/perm buffer on ice for 20 min and then stained with the intracellular marker allophycocyanin (APC)-anti-IFN-γ (BD Pharmingen 554702). After two washes, cells were resuspended in 4% paraformaldehyde (PFA) FACS wash buffer for flow cytometry (BD Influx). PBMCs stimulated with pH1N1 virus were prepared as described above. Samples were analyzed with FlowJo software.

### Tetramer preparation and staining.

H-2D^b^-restricted tetramers of peptides NP_366−374_ and PA_224−233_ and HLA-A*24-restricted tetramers of peptides H1-P25 and H7-P25 were prepared as previously described (30). Preparation and staining of H-2D^b^-restricted tetramers were performed as follows. Briefly, to produce biotinylated peptide-MHC protein, H-2D^b^ was modified by the addition of a substrate sequence for biotinylating enzyme BirA at the C terminus of the α3 domain. *In vitro*-renatured H-2D^b^/peptide complexes were then purified and biotinylated by incubation with d-biotin, ATP, and the biotin protein ligase BirA (Avidity) at 4°C for 12 h. The biotinylated H-2Db was further purified using a Superdex 200 10/300 GL gel-filtration column (GE Healthcare) to remove excess biotin and then mixed with PE-streptavidin (Sigma). Cells from the subjects were stained with PE-tetramer, FITC-conjugated anti-CD3 antibody, and PerCP-cy5.5 anti-CD8 antibody. All samples were analyzed with a FACSCalibur flow cytometer (Becton, Dickinson) after staining.

### Mice and influenza virus infection.

Six-to-8-week-old female C57BL/6 mice were used for virus infections. For the primary infection, mice were lightly anesthetized by inhalation of Drikold and intranasally (i.n.) infected with 10^3.8^ 50% tissue culture-infective doses (TCID_50_) of pH1N1 in 40 µl of phosphate-buffered saline (PBS). The same operation was performed on the control group of mice with 40 µl of PBS. For the secondary challenge, mice were challenged with a high dose of H1N1 (10^5.8^ TCID_50_) and a lethal dose of H5N1 (10^5.2^ TCID_50_) or H7N9 (10^5.6^ TCID_50_) or with PBS 4 weeks later and grouped as H1N1-PBS, H1N1-H1N1, H1N1-H5N1, H1N1-H7N9, PBS-PBS, PBS-H1N1, PBS-H5N1, and PBS-H7N9. Inoculated animals were assessed daily. The mice with severe manifestations after the virus challenge (>20% weight loss plus severe clinical impairment) were humanely euthanized according to our approved protocol.

### Tissue sampling and cell preparation.

Three mice from each group were euthanized at 0, 3, 7, and 14 dpi after secondary challenge. Lungs were collected, weighed, and homogenized in 1 ml of cold DMEM using an IKA T10 homogenizer under sterile conditions. Then, solid debris was pelleted by centrifugation at 5,000 × *g* for 10 min, and the homogenates were used for virus titrations in MDCK cells. Splenocytes were filtered through cell strainers and were lysed with 0.83% ammonium chloride lysis solution to remove erythrocytes (54).

### Immunoinformatics.

Human host and mouse B6 host MHC-I T-cell epitopes of internal proteins of influenza A viruses were downloaded from IEDB (55). Epitopes with peptide lengths of <12 residues were selected, and their positions in proteins were renumbered using isolate A/California/04/2009 as a reference. After merging of duplicates was performed, 129 unique human-host MHC-I T-cell epitopes and 122 unique mice B6-host MHC-I T-cell epitopes were obtained (see Table S2 and S6). Protein sequences of representative strains were downloaded from the GISAID EPIFLU database, and 38 strains were analyzed (see Table S7). Subsequently, multiple-sequence alignment was performed for each protein with the alignment tool MUSCLE v3.8.31 (56). Peptides were extracted as predicted T-cell epitopes of the representative sequences according to the unique epitopes mentioned above. The numbers of different amino acids of each predicted T-cell epitope compared with those from strain A/California/04/2009 were counted. The maximum-likelihood phylogenetic trees for full-length proteins and epitope joint sequences were constructed using Molecular Evolutionary Genetics Analysis MEGA6 software (57) with the JTT model and 1,000 bootstrap replicates.

10.1128/mBio.01408-18.9 TABLE S6  Mouse epitopes in use. Download TABLE S6, DOCX file, 0.1 MB. Copyright © 2018 Zhao et al. 2018 Zhao et al. This content is distributed under the terms of the Creative Commons Attribution 4.0 International license.

10.1128/mBio.01408-18.10 TABLE S7  Strains in use. Download TABLE S7, DOCX file, 0.1 MB. Copyright © 2018 Zhao et al. 2018 Zhao et al. This content is distributed under the terms of the Creative Commons Attribution 4.0 International license.

### Protein expression, refolding, and purification.

The ectodomain of the HLA-A*2402 heavy chain and human β_2_ microglobulin (β_2_m) were expressed in Escherichia coli as inclusion bodies and subsequently refolded *in vitro* in the presence of different peptides. Briefly, the dissolved HLA heavy chain, β_2_m inclusion body, and peptides were diluted at a molar ratio of 1:1:3, respectively, into a refolding buffer (100 mM Tris-HCl, 400 mM l-arginine, 2 mM EDTA-Na, 5 mM glutathione [GSH], 0.5 mM glutathione disulfide [GSSG]) and slowly stirred for 12 h at 4°C. The refolded complexes were then concentrated and purified by Superdex 200 10/300 GL (GE Healthcare) chromatography, further purified on an ion-exchange Resource Q column (GE Healthcare), and manipulated for crystal screening.

### Thermal stability assay.

The stability of each HLA/peptide complex was tested using circular dichroism (CD) spectroscopy. All complexes were refolded and purified as described above and measured at 150 µg/ml in a solution consisting of 20 mM Tris-HCl (pH 8.0) and 50 mM NaCl. CD spectra at 218 nm were measured on a Chirascan spectrometer (Applied Photophysics) using a thermostatically controlled cuvette at temperature intervals of 0.1°C at a rate of 1°C/min between 25 and 90°C. The denaturation curves were generated by nonlinear fitting with origin 8.0 software.

### Crystallization, data collection, and structure determination.

Crystals of HLA-A*2402/H1-P25 and HLA-A*2402/H7-P25 were grown by the sitting-drop, vapor diffusion method at 18°C with a protein/reservoir drop ratio of 1:1 and at a concentration of 10 mg/ml in a mixture containing 20 mM Tris-HCl (pH 8.0) and 150 mM NaCl. The HLA-A*2402/H1-P25 complex crystal grew in a mixture of 0.1 M imidazole (pH 7.0) and 12% (wt/vol) polyethylene glycol 20000. The HLA-A*2402/H7-P25 complex crystal grew in a reaction mixture containing 0.1 M MES (morpholineethanesulfonic acid) monohydrate (pH 6.0) and 14% (wt/vol) polyethylene glycol 4000. Reservoir solutions containing 20% glycerol were used for cryoprotection. The X-ray diffraction data were collected at the Shanghai Synchrotron Radiation Facility (SSRF) 17U beamline. Data were indexed and scaled using DENZO and the HKL2000 software package. The structures were determined using molecular replacement with the program CNS with the structure of Protein Data Bank (PDB) code 3I6L as the model. Extensive model building was performed by hand using COOT, and restrained refinement was performed using REFMAC5. Additional rounds of refinement were performed using the phenix.refine program implemented in the PHENIX software package with anisotropic displacement parameter (ADP) refinement and bulk solvent modeling. The stereochemical quality of the final model was assessed with the program PROCHECK.

The structure-based antigenic analyses of HA proteins pH1N1, H5N1, H6N1, H7N9, and H9N2 were performed using the structures with PDB codes 4JTV for H1N1 (58), 3ZNK for H5N1 (59), 4YY9 for H6N1 (55), 4KOL for H7N9 (60), and 1JSD for H9N2 (61). Structure-related figures were generated using PyMOL (http://www.pymol.org/).

### Statistical analysis.

One-way analysis of variance (ANOVA) was used in multiple comparisons. The two-tailed Student’s *t* test was used to compare data that were normally distributed and the Mann-Whitney test for nonparametric analyses. Asterisks in each figure indicate statistical significance (*, *P* < 0.05; **, *P* < 0.01; ***, *P* < 0.001; ****, *P* < 0.0001). Analyses were performed with GraphPad Prism 6 software (GraphPad Software, Inc., La Jolla, CA).

### Accession number(s).

The coordinates and structure factors of peptides complexed to HLA-A*2402 have been deposited in the Protein Data Bank under accession codes 5WWU (HLA-A*2402/H1-P25), 5WXD (HLA-A*2402/H7-P25, data set 1), and 5WXC (HLA-A*2402/H7-P25, data set 2).
