# Supplementary material for: Heterosubtypic Protections against Human-Infecting Avian Influenza Viruses Correlate to Biased Cross-T-Cell Responses
Source: mBio. 2018 Aug 7;9(4):e01408-18. doi: 10.1128/mBio.01408-18 (PMC6083907; doi:10.1128/mBio.01408-18)
Supplement: TABLE S1 [file mbo004184007st1.docx]

**Table S1. Donor information.**

| **ID** | **Gender** | **Age** | **HLA-A type** | **PBMC** | **Serum** |
| --- | --- | --- | --- | --- | --- |
| 1 | M | 28 | A*24:02 A*30:01 | + | + |
| 2 | F | 22 | N/A | + | + |
| 3 | M | 31 | A*1101 A*1101 | + | + |
| 4 | M | 29 | A*11:01:01 A*24:02:01:01 | + | + |
| 5 | M | 29 | A*1101 A*2402 | + | + |
| 6 | M | 25 | A*02:01, A*03:01 | + | + |
| 7 | F | 24 | A*02:10 A*30:01 | + | + |
| 8 | M | 24 | A*24:02 A*24:02 | + | + |
| 9 | F | 30 | A*24:02 A*33:03 | + | + |
| 10 | F | 25 | A*24:02 A*33:03 | + | + |
| 11 | F | 25 | A*24:02 A*33:03 | + | + |
| 12 | M | 23 | A*02 A*33 | + | + |
| 13 | M | 25 | A*02:01 A*11:01 | + | + |
| 14 | M | 28 | A*02:01 A*02:01 | + | + |
| 15 | F | 25 | A*31:01 A*33:03 | + | + |
| 16 | F | 25 | A*24:02 A*33:03 | + | + |
| 17 | F | 25 | A*02:07 A*24:02 | + | + |
| 18 | F | 25 | A*02:01 A*24:02 | + | + |
| 19 | F | 24 | A*02:10 A*24:02 | + | + |
| 20 | F | 26 | N/A | + | + |
| 21 | M | 27 | A*24:02 A*33:03 | + | + |
| 22 | M | 24 | A*02:01 A*24:02 | + | + |
| 23 | M | 24 | A*11:01 A*33:03 | + | + |
| 24 | M | 28 | A*11:01 A*68:01 | + | + |
| 25 | M | 28 | A*02:03 A*24:02 | + | + |
| 26 | F | 25 | A*24:02 A*26:01 | + | + |
| 27 | M | 26 | A*03:01 A*11:01 | + | + |
| 28 | F | 23 | A*11:01 A*24:02 | + | + |
| 29 | M | 24 | A*24:03 A*31:01 | + | + |
| 30 | M | 40 | A*11:01 A*30:01 | + | + |
| 31 | M | 31 | A*11:01 A*31:01 | - | + |
| 32 | M | 28 | A*02:07 A*31:01 | - | + |
| 33 | M | 28 | A*01:01 A*03:01 | - | + |
| 34 | M | 25 | A*11:01 A*24:02 | - | + |
| 35 | F | 26 | A*02:01 A*11:01 | - | + |
