# Supplementary figures and images for: Heterosubtypic Protections against Human-Infecting Avian Influenza Viruses Correlate to Biased Cross-T-Cell Responses
Source: mBio. 2018 Aug 7;9(4):e01408-18. doi: 10.1128/mBio.01408-18 (PMC6083907; doi:10.1128/mBio.01408-18)

**A**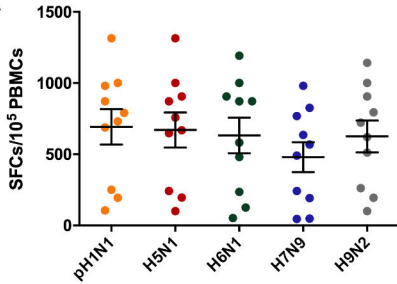**B**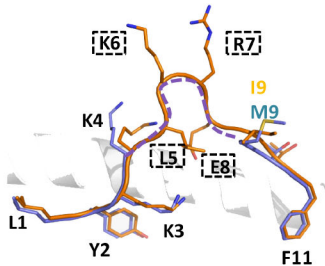

Supplement: FIG S1 [file mbo004184007sf1.pdf]

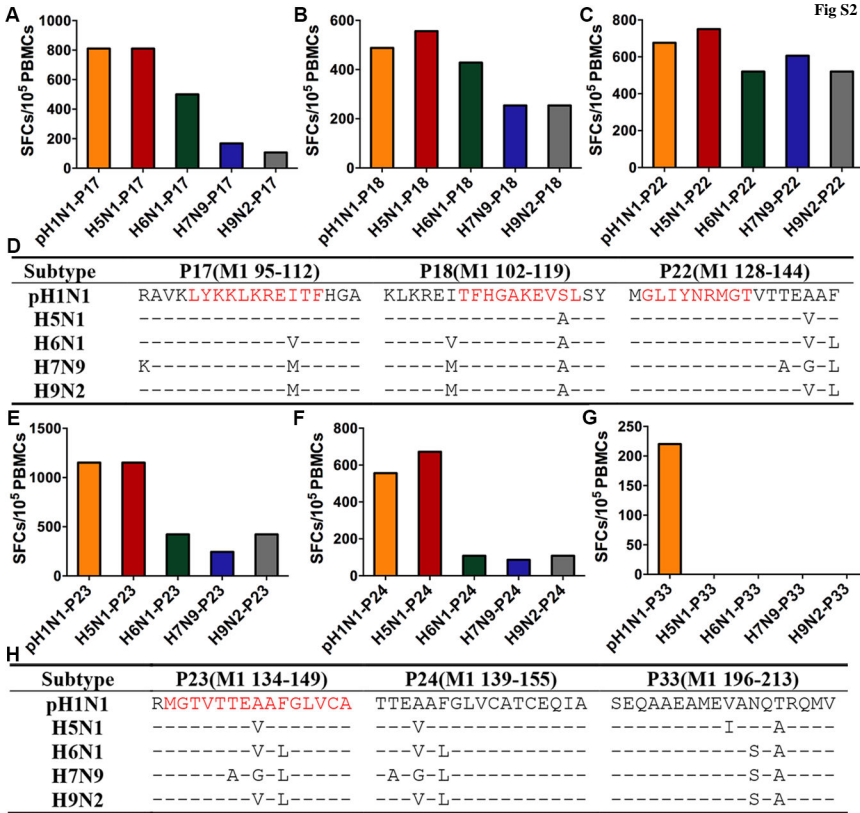

Supplement: FIG S2 [file mbo004184007sf2.pdf]

A

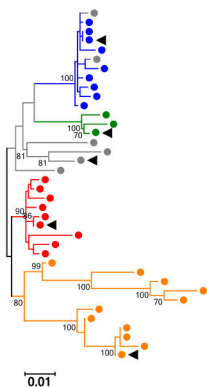

B

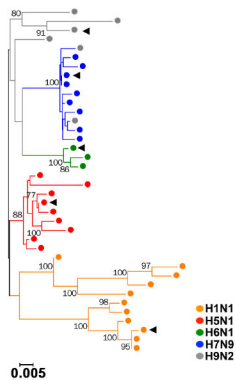

C

Difference VS C404

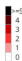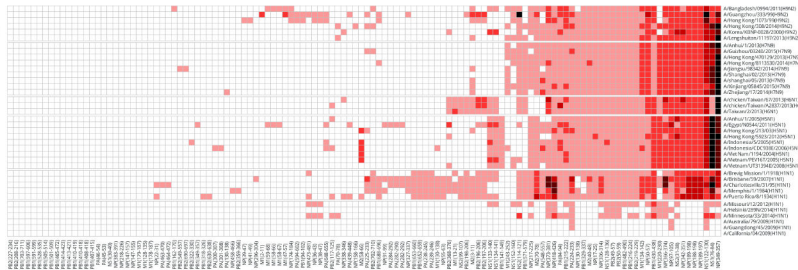

Supplement: FIG S3 [file mbo004184007sf3.pdf]
