# Supplementary material for: Heterosubtypic Protections against Human-Infecting Avian Influenza Viruses Correlate to Biased Cross-T-Cell Responses
Source: mBio. 2018 Aug 7;9(4):e01408-18. doi: 10.1128/mBio.01408-18 (PMC6083907; doi:10.1128/mBio.01408-18)
Supplement: TABLE S2 [file mbo004184007st2.docx]

**Table S2. Human epitopes in use**

| **EpitopeID** | **Protein** | **Peptide** | **Start** | **End** |
| --- | --- | --- | --- | --- |
| 37851 | M1 | LLTEVETYV | 3 | 11 |
| 144494 | M1 | TEVETYVLSI | 5 | 14 |
| 68383 | M2 | VETPIRNEW | 7 | 15 |
| 58567 | M1 | SIIPSGPLK | 13 | 21 |
| 54517 | M1 | RLEDVFAGK | 27 | 35 |
| 144461 | M2 | RLFFKCIYRR | 45 | 54 |
| 33844 | M1 | KTRPILSPLTK | 47 | 57 |
| 27350 | M1 | ILSPLTKGIL | 51 | 60 |
| 20354 | M1 | GILGFVFTL | 58 | 66 |
| 20355 | M1 | GILGFVFTLT | 58 | 67 |
| 20356 | M1 | GILGFVFTLTV | 58 | 68 |
| 27066 | M1 | ILGFVFTLTV | 59 | 68 |
| 97397 | M1 | ILGFVFTL | 59 | 66 |
| 33447 | M2 | KSMREEYRK | 70 | 78 |
| 97488 | M1 | LYRKLKREITF | 99 | 109 |
| 176670 | M1 | TFHGAKEVSL | 108 | 117 |
| 97192 | M1 | ALASCMGLIY | 123 | 132 |
| 4349 | M1 | ASCMGLIY | 125 | 132 |
| 144210 | M1 | ASCMGLIYNR | 125 | 134 |
| 20944 | M1 | GLIYNRMGA | 129 | 137 |
| 54888 | M1 | RMGAVTTEV | 134 | 142 |
| 28309 | M1 | IRHENRMVL | 173 | 181 |
| 54953 | M1 | RMVLASTTAK | 178 | 187 |
| 144471 | M1 | SEQAAEAMEV | 196 | 205 |
| 97161 | M1 | AAEAMEVA | 199 | 206 |
| 97172 | M1 | AEAMEVA | 200 | 206 |
| 97452 | M1 | LKNDLLENLQ | 229 | 238 |
| 175777 | M1 | AYQKRMGVQM | 239 | 248 |
| 19421 | NP | GERQNATEI | 17 | 25 |
| 18406 | NP | FYIQMCTEL | 39 | 47 |
| 7136 | NP | CTELKLSDY | 44 | 52 |
| 32157 | NP | KLSDYEGRL | 48 | 56 |
| 54592 | NP | RLIQNSITI | 55 | 63 |
| 97614 | NP | RMVLSAFDER | 65 | 74 |
| 181219 | NP | KTGGPIYRR | 91 | 99 |
| 34318 | NP | KWMRELVLY | 103 | 111 |
| 24819 | NP | HSNLNDATY | 140 | 148 |
| 145821 | NP | HSNLNDATYQR | 140 | 150 |
| 66924 | NP | TTYQRTRAL | 146 | 154 |
| 21255 | NP | GMDPRMCSL | 158 | 166 |
| 38688 | NP | LPRRSGAAGA | 172 | 181 |
| 55738 | NP | RRSGAAGAAVK | 174 | 184 |
| 65271 | NP | TMVMELVRMIK | 188 | 198 |
| 42974 | NP | MVMELIRMI | 189 | 197 |
| 53890 | NP | RGINDRNFW | 199 | 207 |
| 97772 | NP | YERMCNILKG | 219 | 228 |
| 181251 | NP | YERMCNIL | 219 | 226 |
| 27126 | NP | ILKGKFQTA | 225 | 233 |
| 984 | NP | AEIEDLIFL | 251 | 259 |
| 97173 | NP | AEIEDLIFLA | 251 | 260 |
| 11392 | NP | EDLTFLARS | 254 | 262 |
| 36516 | NP | LIFLARSAL | 256 | 264 |
| 27283 | NP | ILRGSVAHK | 265 | 273 |
| 33285 | NP | KSCLPACVY | 273 | 281 |
| 6615 | NP | CLPACVYGL | 275 | 283 |
| 97583 | NP | QLVWMACHSAA | 327 | 337 |
| 97162 | NP | AAFEDLRVL | 336 | 344 |
| 15500 | NP | FEDLRVLS | 338 | 345 |
| 97298 | NP | FEDLRVSSF | 338 | 346 |
| 56355 | NP | RVLSFIKGTK | 342 | 351 |
| 164335 | NP | QLSTRGVQI | 357 | 365 |
| 3078 | NP | AMDSNTLEL | 373 | 381 |
| 35589 | NP | LELRSRYWA | 379 | 387 |
| 35590 | NP | LELRSRYWAI | 379 | 388 |
| 13263 | NP | ELRSRYWAI | 380 | 388 |
| 39214 | NP | LRSRYWAI | 381 | 388 |
| 60867 | NP | SRYWAIRTR | 383 | 391 |
| 56681 | NP | RYWAIRTR | 384 | 391 |
| 145805 | NP | GQISIQPTFS | 404 | 413 |
| 144488 | NP | SVQPTFSVQR | 407 | 416 |
| 144489 | NP | SVQRNLPFER | 413 | 422 |
| 38469 | NP | LPFDRTTVM | 418 | 426 |
| 176373 | NP | PFERATVMAAF | 419 | 429 |
| 144292 | NP | FQGRGVFEL | 458 | 466 |
| 97526 | NP | NPIVPSFDM | 473 | 481 |
| 2014 | NS1 | AIMDKNIIL | 122 | 130 |
| 19312 | NS1 | GEISPLPSL | 158 | 166 |
| 27467 | NS1 | IMDKNIILKA | 123 | 132 |
| 144293 | NS1 | FQVDCFLWHV | 9 | 18 |
| 97195 | NS2 | ALQLLLEV | 102 | 109 |
| 97405 | NS2 | ITFMQALQLL | 97 | 106 |
| 144440 | NS2 | QEIRTFSFQL | 111 | 120 |
| 1166 | PA | AESRKLLLI | 660 | 668 |
| 6183 | PA | CELTDSSWI | 415 | 423 |
| 17119 | PA | FMYSDFHFI | 46 | 54 |
| 59069 | PA | SLENFRAYV | 225 | 233 |
| 62180 | PA | SVKEKDMTK | 601 | 609 |
| 76533 | PA | YYLEKANKI | 130 | 138 |
| 97176 | PA | AEKPKFLPDLY | 100 | 110 |
| 97579 | PA | QLMWALGENMA | 365 | 375 |
| 97625 | PA | RTMAWTVVNSI | 84 | 94 |
| 144377 | PA | KFLPDLYDYK | 104 | 113 |
| 176343 | PA | LYASPQLEGF | 649 | 658 |
| 177367 | PA | YINTALLNA | 464 | 472 |
| 4177 | PB1 | ARLGKGYMF | 349 | 357 |
| 97458 | PB1 | LLFLKVPA | 7 | 14 |
| 75755 | PB1 | YSHGTGTGY | 30 | 38 |
| 10514 | PB1 | DTVNRTHQY | 41 | 49 |
| 16681 | PB1 | FLKDVMESM | 166 | 174 |
| 165648 | PB1 | RRAIATPGM | 238 | 246 |
| 97315 | PB1 | FVEALARSI | 254 | 262 |
| 97693 | PB1 | TLARSICEK | 257 | 265 |
| 6174 | PB1 | CEKLEQSGL | 263 | 271 |
| 97586 | PB1 | QPEWFRNIL | 329 | 337 |
| 32289 | PB1 | KMARLGKGY | 347 | 355 |
| 97300 | PB1 | FESKSMKL | 357 | 364 |
| 42143 | PB1 | MMMGMFNML | 407 | 415 |
| 97309 | PB1 | FNMLSTVLGV | 412 | 421 |
| 45001 | PB1 | NMLSTVLGV | 413 | 421 |
| 144383 | PB1 | KLVGINMSKK | 471 | 480 |
| 22647 | PB1 | GTFEFTSFFY | 488 | 497 |
| 63635 | PB1 | TFEFTSFFY | 489 | 497 |
| 97299 | PB1 | FEFTSFFY | 490 | 497 |
| 144297 | PB1 | FYRYGFVANF | 496 | 505 |
| 124692 | PB1 | RYGFVANF | 498 | 505 |
| 97314 | PB1 | FVANFSMEL | 501 | 509 |
| 17780 | PB1 | FSMELPSFGV | 505 | 514 |
| 97469 | PB1 | LPSFGVSGI | 509 | 517 |
| 177335 | PB1 | TVIKTNMI | 528 | 535 |
| 21574 | PB1 | GPATAQMAL | 540 | 548 |
| 65880 | PB1 | TQIQTRRSF | 566 | 574 |
| 40571 | PB1 | LVSDGGPNLY | 590 | 599 |
| 70898 | PB1 | VSDGGPNLY | 591 | 599 |
| 97174 | PB1 | AEIMKICST | 741 | 749 |
| 144475 | PB2 | SFSFGGFTFK | 322 | 331 |
| 144526 | PB2 | VLRGFLILGK | 690 | 699 |
| 164387 | PB2 | SRTREILTK | 14 | 22 |
| 177212 | PB2 | PVAGGTSSIYI | 219 | 229 |
| 243963 | PB2 | ILPDMTPSI | 463 | 471 |
