# Supplementary material for: Heterosubtypic Protections against Human-Infecting Avian Influenza Viruses Correlate to Biased Cross-T-Cell Responses
Source: mBio. 2018 Aug 7;9(4):e01408-18. doi: 10.1128/mBio.01408-18 (PMC6083907; doi:10.1128/mBio.01408-18)
Supplement: TABLE S3 [file mbo004184007st3.docx]

**Table S3. Eleven key epitopes showed conservative in H1 and H5 subtypes**

| **StrainName** | **PA**  **(415-423)** | **NP**  **(404-413)** | **PA**  **(601-609)** | **M1**  **(239-248)** | **PB1**  **(41-49)** | **PB1**  **(566-574)** | **PB1**  **(166-174)** | **PB1**  **(254-262)** | **PB1**  **(257-265)** | **NP**  **(44-52)** | **M1**  **(99-109)** |
| --- | --- | --- | --- | --- | --- | --- | --- | --- | --- | --- | --- |
| **MHC restriction** | HLA-B*40：01  HLA-B*40：02  HLA-B*44：02  HLA-B*44：03 |  | HLA-A*03：01  HLA-B*15:01 | HLA-A*24:02 | HLA-A*26:01 | HLA-B*15:01  HLA-B*07:02  HLA-B*27:05 | HLA-A*02:01  HLA-A*26:01  HLA-B*07:02  HLA-B*15:01  HLA-B*08:01 | HLA-A*02:02  HLA-A*02:06 | HLA-A*03:01  HLA-A*11:01  HLA-A*68:01 | HLA-A*01:01 | HLA-A*24:02 |
| A/California/04/2009(H1N1) | CELTDSSWI | GQISVQPTFS | SVKEKDMTK | AYQKRMGVQM | DTVNRTHQY | TQIQTRRSF | FLKDVMESM | FVETLARSI | TLARSICEK | CTELKLSDY | LYKKLKREITF |
| A/Guangdong/45/2009(H1N1) | ......... | .......... | ......... | .......... | ......... | ......... | ......... | ......... | ......... | ......... | ........... |
| A/Helsinki/289N/2014(H1N1) | ......... | .......... | ......... | .......... | ......... | ......... | ......... | ......... | ......... | ......... | ........... |
| A/Australia/79/2009(H1N1) | ......... | .......... | ......... | .......... | ......... | ......... | ......... | ......... | ......... | ......... | ........... |
| A/Missouri/12/2012(H1N1) | ......... | .......... | ......... | .......... | ......... | ......... | ......... | ......... | ......... | ......... | ........... |
| A/Minnesota/33/2014(H1N1) | ......... | .......... | ......... | .......... | ......... | ......... | ......... | ......... | ......... | ......... | ........... |
| A/Brevig_Mission/1/1918(H1N1) | ......... | .......... | ......... | .......... | ......... | ......... | ......... | ......... | ......... | ......... | ..R........ |
| A/Puerto_Rico/8/1934(H1N1) | ......... | ....I..... | ......... | .......... | ......... | ......... | ......... | ......... | ......... | ......... | ..R........ |
| A/Memphis/1/1984(H1N1) | ......I.. | ....I..... | ......... | .......... | ......... | ......... | ......D.. | ......... | ......... | ......N.. | ..R........ |
| A/Brisbane/59/2007(H1N1) | ......I.. | ....T..... | ......... | .......... | ......... | ......... | ......... | ......... | ......... | ......N.. | ..R........ |
| A/Charlottesville/31/95(H1N1) | ......I.. | ....I..... | ......... | .......... | ......... | ......... | ......... | ......... | ......... | ......N.. | ..R........ |
| A/Hong_Kong/5923/2012(H5N1) | ......... | .......... | ......... | .......... | ......... | ......... | ......... | ......... | ......... | ........H | ........... |
| A/Anhui/1/2005(H5N1) | ......... | .......... | ......... | .......... | ......... | ......... | ......... | ......... | ......... | ......... | ........... |
| A/Vietnam/UT31394II/2008(H5N1) | ......... | .......... | ......... | .......... | ......... | ......... | ......... | .......N. | ....N.... | ......... | ........... |
| A/Hong_Kong/213/03(H5N1) | ......... | .......... | ......... | .......... | ......... | ......... | ......... | ......... | ......... | ......... | ........... |
| A/Vietnam/PEV16T/2005(H5N1) | ......... | .......... | ......... | T......... | ......... | ......... | ......... | ......... | ......... | ......... | ........... |
| A/Viet_Nam/1194/2004(H5N1) | ......... | .......... | ......... | .......... | ......... | ......... | ......... | ......... | ......... | ......... | ........... |
| A/Egypt/N0544/2011(H5N1) | ......... | ....I..... | ......... | .......... | ......... | ......... | ......... | ......... | ......... | ......... | ........... |
| A/Indonesia/CDC938E/2006(H5N1) | ......... | .......... | ......... | .......... | ......... | ......... | ......... | ......... | ......... | ......... | ........... |
| A/Indonesia/5/2005(H5N1) | ......... | .......... | ......... | .......... | ......... | ......... | ......... | ......... | ......... | ......... | ........... |
| A/Korea/KBNP-0028/2000(H9N2) | ......... | .......... | ......... | ...R...... | ......... | ......... | ......G.. | ...H..... | H........ | ........H | ..R........ |
| A/Guangzhou/333/99(H9N2) | ......... | .......... | .I....... | .......... | ......... | ......... | ......... | ...A..... | A........ | ........H | ........M.. |
| A/Hong_Kong/1073/99(H9N2) | ......... | .......... | .I....... | .......... | ......... | ......... | ......... | ...A..... | A........ | ........Q | ........M.. |
| A/Bangladesh/0994/2011(H9N2) | ......... | .......... | ......... | .......L.. | ......... | ......... | X........ | .......T. | ....T.... | ........Q | ........M.. |
| A/chicken/Taiwan/A2837/2013(H6N1) | ......... | .......... | ......... | .......... | ......... | S........ | ......... | .......N. | ....N.... | ........N | ........V.. |
| A/Taiwan/2/2013(H6N1) | ......... | .......... | ......... | .......... | ......... | ......... | ......... | ......... | ......... | ........N | ........V.. |
| A/chicken/Taiwan/67/2013(H6N1) | ......... | .......... | ......... | .......... | ......... | ......... | ......... | ......... | ......... | ........N | ........V.. |
| A/Jiangsu/98342/2014(H7N9) | ........V | ..V....... | ......L.. | ...N.....L | .......K. | .......A. | ......D.. | ...A..... | A........ | ........N | ........M.. |
| A/shanghai/05/2013(H7N9) | ........V | ..V....... | ..R...L.. | ...N.....L | .......K. | .......A. | ......D.. | ...A..... | A........ | ........N | ........M.. |
| A/Xinjiang/05845/2015(H7N9) | ........V | ..V....... | ..R...L.. | ...N.....L | .......K. | .......A. | ......D.. | ...A..... | A........ | ........N | ........M.. |
| A/Zhejiang/17/2014(H7N9) | ........V | ..V....... | ......L.. | ...N.....L | .......K. | .......A. | ......D.. | ...A..... | A........ | ........N | ........M.. |
| A/Lengshuitan/11197/2013(H9N2) | ........V | ....I..... | ......L.. | ...N.....L | .......K. | .......A. | .....VD.. | ...A..... | A........ | ........N | ........M.. |
| A/Guizhou/03240/2015(H7N9) | ........V | ....I..... | ......L.. | ...N.....L | .......K. | .......A. | ......D.V | ...A..... | A........ | ........N | ........M.. |
| A/Hong_Kong/8113530/2014(H7N9) | ........V | ..V....... | ......L.. | ...N...... | .......K. | .......A. | .....VD.. | ...A..... | A........ | ........N | ........M.. |
| A/Hong_Kong/308/2014(H9N2) | ........V | ..V....... | ......L.. | ...N.....L | .......K. | .......A. | ......D.. | ...A..... | A........ | ........N | ........M.. |
| A/Hong_Kong/470129/2013(H7N9) | ........V | ..V....... | ......L.. | ...N.....L | .......K. | .......A. | ......D.. | ...A..... | A........ | ........N | ........M.. |
| A/Shanghai/02/2013(H7N9) | ........V | ..V....... | ......L.. | ...N.....L | .......K. | .......A. | ......D.. | ...A..... | A........ | ........N | ........M.. |
| A/Anhui/1/2013(H7N9) | ........V | ..V....... | ......L.. | ...N.....L | .......K. | .......A. | ......D.. | ...A..... | A........ | ........N | ........M.. |
