# Supplementary material for: Heterosubtypic Protections against Human-Infecting Avian Influenza Viruses Correlate to Biased Cross-T-Cell Responses
Source: mBio. 2018 Aug 7;9(4):e01408-18. doi: 10.1128/mBio.01408-18 (PMC6083907; doi:10.1128/mBio.01408-18)
Supplement: TABLE S4 [file mbo004184007st4.docx]

**Table S4. Data collection and refinement statistics**

|  | HLA-A*2402 /H1-P25 | HLA-A*2402 /H7-P25 (data set 1) | HLA-A*2402 /H7-P25 (data set 2) |
| --- | --- | --- | --- |
| **Data collection** |  |  |  |
| Space Group | P2_1_2_1_2_1_ | P2_1_2_1_2_1_ | P2_1_ |
| Cell parameters (Å) | a=71.752  b=79.099  c=88.100  α=90.0  β=90.0  γ=90.0 | a=67.470  b=78.983  c=88.052  α=90.0  β=90.0  γ=90.0 | a=81.373  b=67.292  c=88.346  α=90.0  β=98.430  γ=90.0 |
| Wavelength (Å ) | 0.97774 | 1.03871 | 0.97930 |
| Resolution (Å) | 50.0-2.8  (2.9-2.8) ^a^ | 50.0- 3.30  (3.42- 3.30) | 50.0-2.3  (2.38-2.30) |
| Total reflections | 138842 | 52215 | 202325 |
| Completeness (%)^b^ | 100 (100) | 100 (100) | 99.8 (99.1) |
| R_merge_ (%)^c^ | 12.5(77.2) | 15.8(83.1) | 9.1(56.9) |
| I/σ | 16.8(2.6) | 12.2(2.5) | 16.8(4.1) |
| **Refinement:** |  |  |  |
| R_factor_ (%)^d^ | 22.5 | 20.3 | 17.7 |
| R_free_ (%) | 28.4 | 25.4 | 22.4 |
| r.m.s. deviation |  |  |  |
| Bonds (Å) | 0.003 | 0.003 | 0.006 |
| Angles (°) | 0.703 | 0.681 | 0.855 |
| Ramachandran map^e^ |  |  |  |
| Favored (%) | 98.0 | 96.0 | 98.0 |
| Allowed (%) | 2.0 | 4.0 | 2.0 |
| Disallowed (%) | 0.0 | 0.0 | 0.0 |

^a^Values in parentheses refer to statistics in the outermost resolution shell.

^b^Data completeness = (no. of independent reflections)/(total theoretical number).

^c^R_merge_=∑_hkl_∑_i_ ∣I_i_ -〈I〉∣∑_hkl_∑_i_I_i_, where I_i_ is the observed intensity and 〈I〉 is the average intensity of multiple observations of symmetry related reflections.

^d^R=∑_hkl_ ‖F_obs_∣- k∣F_cal_ ∣∣/ ∑_hkl_ ∣F_obs_∣, where R_free_ is calculated for a randomly chosen 5% of reflections, R_work_ is calculated for the remaining 95% of reflections used for structure refinement.

^e^Ramachandran plots were generated by using the PROCHECK program of the CCP4i suite.
