# Supplementary material for: Heterosubtypic Protections against Human-Infecting Avian Influenza Viruses Correlate to Biased Cross-T-Cell Responses
Source: mBio. 2018 Aug 7;9(4):e01408-18. doi: 10.1128/mBio.01408-18 (PMC6083907; doi:10.1128/mBio.01408-18)
Supplement: TABLE S5 [file mbo004184007st5.docx]

**Table S5. M1 overlapping peptide pools of influenza viruses**

| Name | Residues | Positions | A/California/04/2009  (H1N1) | A/Vietnam/1194/2004  (H5N1) | A/Taiwan/02/2013  (H6N1) | A/Anhui/01/2013  (H7N9) | A/Hong Kong/1073/1999  (H9N2) |
| --- | --- | --- | --- | --- | --- | --- | --- |
| P2 | 15 | 1-15 | MSLLTEVETYVLSII | - | MSLLTEVETYVLSVV | - | - |
| P3 | 17 | 5-21 | TEVETYVLSIIPSGPLK | - | TEVETYVLSVVPSGPLK | - | - |
| P4 | 18 | 11-28 | VLSIIPSGPLKAEIAQRL | VLSIIPSGPLKAEIAQKL | VLSVVPSGPLKAEIAQRL | - | - |
| P5 | 18 | 18-35 | GPLKAEIAQRLESVFAGK | GPLKAEIAQKLEDVFAGK | GPLKAEIAQRLEDVFAGK | GPLKAEIAQRLEDVFAGK | GPLKAEIAQRLEDVFAGK |
| P6 | 18 | 25-42 | AQRLESVFAGKNTDLEAL | AQKLEDVFAGKNTDLEAL | AQRLEDVFAGKNTDLEAL | AQRLEDVFAGKNADLEAL | AQRLEDVFAGKNTDLEAL |
| P7 | 18 | 32-49 | FAGKNTDLEALMEWLKTR | - | - | FAGKNADLEALMEWIKTR | - |
| P8 | 17 | 39-55 | LEALMEWLKTRPILSPL | - | - | LEALMEWIKTRPILSPL | - |
| P9 | 18 | 45-62 | WLKTRPILSPLTKGILGF | - | - | WIKTRPILSPLTKGILGF | - |
| P15 | 18 | 81-98 | QNALNGNGDPNNMDRAVK | - | - | QNALNGNGDPNNMDKAVK | - |
| P16 | 18 | 88-105 | GDPNNMDRAVKLYKKLKR | - | - | GDPNNMDKAVKLYKKLKR | - |
| P17 | 18 | 95-112 | RAVKLYKKLKREITFHGA | - | RAVKLYKKLKREVTFHGA | KAVKLYKKLKREMTFHGA | RAVKLYKKLKREMTFHGA |
| P18 | 18 | 102-119 | KLKREITFHGAKEVSLSY | KLKREITFHGAKEVALSY | KLKREVTFHGAKEVALSY | KLKREMTFHGAKEVALSY | KLKREMTFHGAKEVALSY |
| P19 | 16 | 109-124 | FHGAKEVSLSYSTGAL | FHGAKEVALSYSTGAL | FHGAKEVALSYSAGAL | FHGAKEVALSYSTGAL | FHGAKEVALSYSTGAL |
| P20 | 18 | 114-131 | EVSLSYSTGALASCMGLI | EVALSYSTGALASCMGLI | EVALSYSAGALSGCMGLI | EVALSYSTGALASCMGLI | EVALSYSTGALASCMGLI |
| P21 | 18 | 121-138 | TGALASCMGLIYNRMGTV | - | AGALSGCMGLIYNRMGTV | - | - |
| P22 | 17 | 128-144 | MGLIYNRMGTVTTEAAF | MGLIYNRMGTVTTEVAF | MGLIYNRMGTVTTEVAL | MGLIYNRMGTVTAEGAL | MGLIYNRMGTVTTEVAL |
| P23 | 16 | 134-149 | RMGTVTTEAAFGLVCA | RMGTVTTEVAFGLVCA | RMGTVTTEVALGLVCA | RMGTVTAEGALGLVCA | RMGTVTTEVALGLVCA |
| P24 | 17 | 139-155 | TTEAAFGLVCATCEQIA | TTEVAFGLVCATCEQIA | TTEVALGLVCATCEQIA | TAEGALGLVCATCEQIA | TTEVALGLVCATCEQIA |
| P25 | 16 | 145-160 | GLVCATCEQIADSQHR | - | - | GLVCATCEQIADAQHR | GLVCATCEQIADAQHR |
| P26 | 17 | 150-166 | TCEQIADSQHRSHRQMA | - | - | TCEQIADAQHRSHRQMA | TCEQIADAQHRSHRQMA |
| P27 | 18 | 156-173 | DSQHRSHRQMATTTNPLI | DSQHRSHRQMATITNPLI | DSQHRSHRQMATITNPLI | DAQHRSHRQMATTTNPLI | DAQHRSHRQMATTTNPLI |
| P28 | 18 | 163-180 | RQMATTTNPLIRHENRMV | RQMATITNPLIRHENRMV | RQMATITNPLIRHENRMV | - | - |
| P32 | 18 | 189-206 | MEQMAGSSEQAAEAMEVA | MEQMAGSSEQAAEAMEIA | - | - | - |
| P33 | 18 | 196-213 | SEQAAEAMEVANQTRQMV | SEQAAEAMEIANQARQMV | SEQAAEAMEVASQARQMV | SEQAAEAMEVASQARQMV | SEQAAEAMEVASQARQMV |
| P34 | 17 | 203-219 | MEVANQTRQMVHAMRTI | MEIANQARQMVQAMRTI | MEVASQARQMVQAMRTI | MEVASQARQMVQAMRTV | MEVASQARQMVQAMRTI |
| P35 | 19 | 209-227 | TRQMVHAMRTIGTHPSSSA | ARQMVQAMRTIGTHPNSSA | ARQMVQAMRTIGTHPSSSA | ARQMVQAMRTVGTHPNSST | ARQMVQAMRTIGTHPSSSA |
| P36 | 18 | 217-234 | RTIGTHPSSSAGLKDDLL | RTIGTHPNSSAGLRDNLL | RTIGTHPSSSAGLRDDLL | RTVGTHPNSSTGLKDDLI | RTIGTHPSSSAGLKDDLI |
| P37 | 18 | 224-241 | SSSAGLKDDLLENLQAYQ | NSSAGLRDNLLENLQAYQ | SSSAGLRDDLLENLQAYQ | NSSTGLKDDLIENLQAYQ | SSSAGLKDDLIENLQAYQ |
| P38 | 18 | 231-248 | DDLLENLQAYQKRMGVQM | - | - | DDLIENLQAYQNRMGVQL | DDLIENLQAYQKRMGVQM |
| P39 | 15 | 238-252 | QAYQKRMGVQMQRFK | - | - | QAYQNRMGVQLQRFK | - |

“-“ represents the same sequence with that of 09pH1N1
