# Supplementary material for: Heterosubtypic Protections against Human-Infecting Avian Influenza Viruses Correlate to Biased Cross-T-Cell Responses
Source: mBio. 2018 Aug 7;9(4):e01408-18. doi: 10.1128/mBio.01408-18 (PMC6083907; doi:10.1128/mBio.01408-18)
Supplement: TABLE S6 [file mbo004184007st6.docx]

**Table S6. Mice epitopes in use**

| **EpitopeID** | **Protein** | **Peptide** | **Start** | **End** |
| --- | --- | --- | --- | --- |
| 41602 | M1 | MGLIYNRM | 128 | 135 |
| 58567 | M1 | SIIPSGPLK | 13 | 21 |
| 36782 | M1 | LIYNRMGAV | 130 | 138 |
| 54888 | M1 | RMGAVTTEV | 134 | 142 |
| 54953 | M1 | RMVLASTTAK | 178 | 187 |
| 42959 | M1 | MVLASTTAK | 179 | 187 |
| 59322 | M1 | SLLTEVETYV | 2 | 11 |
| 22690 | M1 | GTHPSSSAGLK | 220 | 230 |
| 54898 | M1 | RMGVQMQRFK | 243 | 252 |
| 33844 | M1 | KTRPILSPLTK | 47 | 57 |
| 20354 | M1 | GILGFVFTL | 58 | 66 |
| 20,355 | M1 | GILGFVFTLT | 58 | 67 |
| 27066 | M1 | ILGFVFTLTV | 59 | 68 |
| 37850 | M2 | LLTEVETPI | 3 | 11 |
| 153854 | M2 | LWILDRLFF | 40 | 48 |
| 20953 | M2 | GLKGGPSTE | 58 | 66 |
| 33447 | M2 | KSMREEYRK | 70 | 78 |
| 175652 | NP | TAGLTHIMI | 130 | 138 |
| 67436 | NP | TYQRTRALV | 147 | 155 |
| 67440 | NP | TYQRTRALVTG | 147 | 157 |
| 21255 | NP | GMDPRMCSL | 158 | 166 |
| 19421 | NP | GERQNATEI | 17 | 25 |
| 167156 | NP | RRSGAAVAAHK | 174 | 184 |
| 65271 | NP | TMVMELVRMIK | 188 | 198 |
| 42974 | NP | MVMELIRMI | 189 | 197 |
| 42975 | NP | MVMELVRMIK | 189 | 198 |
| 25478 | NP | IAYERMCNI | 217 | 225 |
| 5757 | NP | AYERMCNIL | 218 | 226 |
| 4940 | NP | ATEIRASVGK | 22 | 31 |
| 175669 | NP | YSLVGIDPF | 296 | 304 |
| 521400 | NP | LLQNSQVYS | 306 | 314 |
| 97583 | NP | QLVWMACHSAA | 327 | 337 |
| 15501 | NP | FEDLRVLSF | 338 | 346 |
| 56355 | NP | RVLSFIKGTK | 342 | 351 |
| 22735 | NP | GTKVVPRGK | 349 | 357 |
| 26315 | NP | IGRFYIQM | 36 | 43 |
| 4,581 | NP | ASNENMEAM | 366 | 374 |
| 3078 | NP | AMDSNTLEL | 373 | 381 |
| 53806 | NP | RFYIQMCTEL | 38 | 47 |
| 60,867 | NP | SRYWAIRTR | 383 | 391 |
| 18406 | NP | FYIQMCTEL | 39 | 47 |
| 175601 | NP | IQMCTELKL | 41 | 49 |
| 38,468 | NP | LPFDRTTIM | 418 | 426 |
| 144292 | NP | FQGRGVFEL | 458 | 466 |
| 60089 | NP | SPIVPSFDM | 473 | 481 |
| 57322 | NP | SDYEGRLI | 50 | 57 |
| 54,592 | NP | RLIQNSITI | 55 | 63 |
| 555792 | NP | RTGGPIYRR | 91 | 99 |
| 75671 | NP | YRRVNGKWM | 97 | 105 |
| 2,014 | NS1 | AIMDKNIIL | 122 | 130 |
| 125444 | NS1 | IILKANFSV | 128 | 136 |
| 43931 | NS1 | NFSVIFDR | 133 | 140 |
| 17871 | NS1 | FSVIFDRL | 134 | 141 |
| 11640 | NS1 | EEGAIVGEI | 152 | 160 |
| 103050 | NS1 | APFLDRLRRDQ | 30 | 40 |
| 2667 | NS1 | ALKMTMASV | 76 | 84 |
| 56055 | NS2 | RTFSFQLI | 114 | 121 |
| 41784 | NS2 | MITQFESL | 31 | 38 |
| 515892 | NS2 | FMQALHLLL | 99 | 107 |
| 76533 | PA | YYLEKANKI | 130 | 138 |
| 61,151 | PA | SSLENFRAYV | 224 | 233 |
| 59069 | PA | SLENFRAYV | 225 | 233 |
| 48650 | PA | PNGYIEGK | 238 | 245 |
| 44125 | PA | NGYIEGKL | 239 | 246 |
| 16733 | PA | FLLMDALKL | 282 | 290 |
| 515862 | PA | FLLMDALKLSI | 282 | 292 |
| 37999 | PA | LMDALKLSI | 284 | 292 |
| 12194 | PA | EGIPLYDA | 300 | 307 |
| 20399 | PA | GIPLYDAI | 301 | 308 |
| 175570 | PA | ASMRRNYFTA | 439 | 448 |
| 6244 | PA | CFMYSDFHF | 45 | 53 |
| 17119 | PA | FMYSDFHFI | 46 | 54 |
| 72054 | PA | VYINTALL | 463 | 470 |
| 177367 | PA | YINTALLNA | 464 | 472 |
| 41998 | PA | MLLRSAIGQV | 548 | 557 |
| 59673 | PA | SMIEAESSV | 594 | 602 |
| 59587 | PA | SLYASPQL | 648 | 655 |
| 2711 | PA | ALLKHRFEI | 70 | 78 |
| 97625 | PA | RTMAWTVVNSI | 84 | 94 |
| 62904 | PB1 | TALANTIEV | 141 | 149 |
| 54584 | PB1 | RLIDFLKDV | 162 | 170 |
| 56003 | PB1 | RSYLIRAL | 215 | 222 |
| 62631 | PB1 | SYLIRALTL | 216 | 224 |
| 515835 | PB1 | FLAMITYMT | 318 | 326 |
| 51809 | PB1 | QPEWFRNVL | 329 | 337 |
| 4177 | PB1 | ARLGKGYMF | 349 | 357 |
| 42143 | PB1 | MMMGMFNML | 407 | 415 |
| 522500 | PB1 | MMGMFNMLSTV | 408 | 418 |
| 21267 | PB1 | GMFNMLSTV | 410 | 418 |
| 516896 | PB1 | GMFNMLSTVL | 410 | 419 |
| 45001 | PB1 | NMLSTVLGV | 413 | 421 |
| 42068 | PB1 | MLSTVLGVSI | 414 | 423 |
| 153884 | PB1 | RYTKTTYWW | 430 | 438 |
| 53812 | PB1 | RFYRTCKL | 465 | 472 |
| 62591 | PB1 | SYINRTGTF | 482 | 490 |
| 97314 | PB1 | FVANFSMEL | 501 | 509 |
| 17780 | PB1 | FSMELPSFGV | 505 | 514 |
| 177335 | PB1 | TVIKTNMI | 528 | 535 |
| 55736 | PB1 | RRSFEIKKL | 571 | 579 |
| 70898 | PB1 | VSDGGPNLY | 591 | 599 |
| 44947 | PB1 | NLYNIRNLHI | 597 | 606 |
| 2255 | PB1 | AKNMEYDA | 652 | 659 |
| 32556 | PB1 | KNMEYDAV | 653 | 660 |
| 153858 | PB1 | MYQKCCTLF | 688 | 696 |
| 61497 | PB1 | SSYRRPVGI | 703 | 711 |
| 16566 | PB1 | FLEESHPGI | 94 | 102 |
| 153898 | PB2 | TYFEKVERL | 117 | 125 |
| 187280 | PB2 | CKISPLMVAYM | 196 | 206 |
| 31429 | PB2 | KISPLMVAYM | 197 | 206 |
| 28650 | PB2 | ISPLMVAYM | 198 | 206 |
| 13981 | PB2 | ERELVRKTR | 208 | 216 |
| 177212 | PB2 | PVAGGTSSIYI | 219 | 229 |
| 72047 | PB2 | VYIEVLHL | 227 | 234 |
| 57892 | PB2 | SFSFGGFTF | 322 | 330 |
| 23329 | PB2 | GYEEFTMV | 359 | 366 |
| 55531 | PB2 | RRATAILRK | 368 | 376 |
| 72858 | PB2 | WMMAMKYPI | 49 | 57 |
| 534615 | PB2 | MMAMKYPITA | 50 | 59 |
| 67442 | PB2 | TYQWIIRNW | 549 | 557 |
| 54941 | PB2 | RMQFSSFTV | 630 | 638 |
| 69735 | PB2 | VLRGFLIL | 690 | 697 |
| 33257 | PB2 | KRYGPALSI | 702 | 710 |
