# Supplementary material for: Heterosubtypic Protections against Human-Infecting Avian Influenza Viruses Correlate to Biased Cross-T-Cell Responses
Source: mBio. 2018 Aug 7;9(4):e01408-18. doi: 10.1128/mBio.01408-18 (PMC6083907; doi:10.1128/mBio.01408-18)
Supplement: TABLE S7 [file mbo004184007st7.docx]

**Table S7. Strains in use**

| **Strain Name** |
| --- |
| A/California/04/2009 (H1N1) |
| A/Guangdong/45/2009 (H1N1) |
| A/Helsinki/289N/2014 (H1N1) |
| A/Australia/79/2009 (H1N1) |
| A/Missouri/12/2012 (H1N1) |
| A/Minnesota/33/2014 (H1N1) |
| A/Brevig_Mission/1/1918 (H1N1) |
| A/Puerto_Rico/8/1934 (H1N1) |
| A/Memphis/1/1984 (H1N1) |
| A/Brisbane/59/2007 (H1N1) |
| A/Charlottesville/31/95 (H1N1) |
| A/Hong_Kong/5923/2012 (H5N1) |
| A/Anhui/1/2005 (H5N1) |
| A/Vietnam/UT31394II/2008 (H5N1) |
| A/Hong_Kong/213/03 (H5N1) |
| A/Vietnam/PEV16T/2005 (H5N1) |
| A/Viet_Nam/1194/2004 (H5N1) |
| A/Egypt/N0544/2011 (H5N1) |
| A/Indonesia/CDC938E/2006 (H5N1) |
| A/Indonesia/5/2005 (H5N1) |
| A/Korea/KBNP-0028/2000 (H9N2) |
| A/Guangzhou/333/99 (H9N2) |
| A/Hong_Kong/1073/99 (H9N2) |
| A/Bangladesh/0994/2011 (H9N2) |
| A/chicken/Taiwan/A2837/2013 (H6N1) |
| A/Taiwan/2/2013 (H6N1) |
| A/chicken/Taiwan/67/2013 (H6N1) |
| A/Jiangsu/98342/2014 (H7N9) |
| A/shanghai/05/2013 (H7N9) |
| A/Xinjiang/05845/2015 (H7N9) |
| A/Zhejiang/17/2014 (H7N9) |
| A/Lengshuitan/11197/2013 (H9N2) |
| A/Guizhou/03240/2015 (H7N9) |
| A/Hong_Kong/8113530/2014 (H7N9) |
| A/Hong_Kong/308/2014 (H9N2) |
| A/Hong_Kong/470129/2013 (H7N9) |
| A/Shanghai/02/2013 (H7N9) |
| A/Anhui/1/2013 (H7N9) |
